# ﻿Systematics of the braconid wasp subfamily Rhysipolinae (Hymenoptera, Braconidae) based on UCE data, with the description of a new Neotropical genus

**DOI:** 10.3897/zookeys.1234.147859

**Published:** 2025-04-08

**Authors:** Gerardo Y. García-Acosta, Rubén Castañeda-Osorio, Sergey A. Belokobylskij, Eduardo M. Shimbori, Jovana M. Jasso-Martínez, Angélica M. Penteado-Dias, Alejandro Zaldívar-Riverón

**Affiliations:** 1 Colección Nacional de Insectos, Departamento de Zoología, Instituto de Biología, Universidad Nacional Autónoma de México, 3er circuito exterior s/n, Ciudad Universitaria, Coyoacán, Ciudad de México, Mexico Universidad Nacional Autónoma de México Ciudad de México Mexico; 2 Zoological Institute of the Russian Academy of Sciences, Universitetskaya naberezhnaya 1, Saint Petersburg, Russia Zoological Institute of the Russian Academy of Sciences Saint Petersburg Russia; 3 Departamento de Ecologia e Biologia Evolutiva, Universidade Federal de São Carlos, São Carlos, São Paulo, Brazil Universidade Federal de São Carlos São Paulo Brazil; 4 Centre de coopération internationale en recherche agronomique pour le développement (CIRAD), Centre de Biologie pour la Gestion de Populations (CBGP), Montpellier, F-34398, France Centre de Biologie pour la Gestion de Populations Montpellier France

**Keywords:** Cyclostome, Ichneumonoidea, new genus, phylogeny, ultraconserved elements

## Abstract

Rhysipolinae is a small cosmopolitan cyclostome subfamily of braconid wasps, currently comprising 10 genera and more than 80 species. The two species of the subfamily whose biology has been confirmed are koinobiont ectoparasitoids of lepidopteran larvae, deviating from the two common parasitoid strategies in Braconidae (koinobiont-endoparasitoid, idiobiont-ectoparasitoid). Defining the limits of Rhysipolinae has been challenging due to the lack of exclusive morphological features and difficulties in resolving its phylogenetic relationships based on both morphological and Sanger DNA sequence data. However, recent phylogenomic studies using nuclear ultraconserved elements (UCEs) and mitochondrial genome sequences have begun to clarify its relationships, although various generic boundaries remain unclear. Here a phylogenomic analysis based on UCE data was performed including 32 species of nine rhysipoline genera to assess the monophyly of the subfamily as well as its generic limits. Our phylogenetic analysis confirmed the monophyly of Rhysipolinae, but no unique external morphological features were found for its diagnosis. Most genera were recovered as monophyletic except *Rhysipolis* Förster, 1863, whose clade included *Cerophanes* Tobias, 1971 and *Troporhysipolis* Quicke, Belokobylskij & Butcher, 2016. Based on our molecular and morphological evidence, we synonymise *Cerophanes***syn. nov.** with *Rhysipolis* and describe the new genus and species *Rogapolisnomai* García-Acosta, Shimbori, Castañeda-Osorio & Zaldívar-Riverón **gen. et sp. nov.**, which is mainly characterised by a median longitudinal carina on the second metasomal tergum, a feature previously predominantly occurring in Rogadinae. Moreover, *Pseudavga* Tobias, 1964 **syn. nov.** is proposed as a subgenus of *Pachystigmus* Hellén, 1927. A taxonomic diagnosis for Rhysipolinae and a key to its currently valid genera are also provided.

## ﻿Introduction

Rhysipolinae Belokobylskij, 1984 is a small cosmopolitan cyclostome subfamily of braconid wasps, currently comprising 10 genera and over 80 extant species (Yu et al. 2016; Quicke et al. 2016; Jasso-Martínez et al. 2021, 2022a). Its type genus, *Rhysipolis* Förster, 1863, is the most widespread and speciose, comprising 22 valid species distributed across the Palaearctic, Nearctic, Neotropical, Oriental, and Australasian regions (Yu et al. 2016). While the biology of most rhysipoline species remains unknown, two species with well-documented strategies, *Rh.decorator* (Haliday, 1836) and *Pseudavgaflavicoxa* Tobias, 1964, are known to be koinobiont ectoparasitoids of lepidopteran larvae from the families Crambidae, Gelechiidae, Gracillariidae, Momphidae, and Bucculatricidae (Shaw 1983; Shaw and Sims 2015; Yu et al. 2016; Shaw 2017). This parasitoid strategy represents a notable deviation from the typical koinobiont-endoparasitoid and idiobiont-ectoparasitoid strategies observed in Braconidae (Shaw 1983). It has been proposed that this behaviour might constitute an evolutionary intermediate stage between ectoparasitoid idiobiosis and endoparasitoid koinobiosis (Gauld 1988). Additionally, the biology of three species from other rhysipoline genera, *Cantharoctonus* Viereck, 1912, *Pachystigmus* Hellén, 1927, and *Parachremylus* Granger, 1949 is partially known, with all being reported as ectoparasitoids of lepidopteran larvae (Belokobylskij and Tobias 1986; Whitfield and Wagner 1991; Belokobylskij and Maeto 2006).

The taxonomic definition of Rhysipolinae has historically been challenging due to the absence of exclusive morphological features and the difficulty in consistently delineating its generic limits, composition, and phylogenetic relationships among its genera based on external morphology (Whitfield and van Achterberg 1987; Whitfield 1992; Wharton 1993; Spencer and Whitfield 1999; Scatolini et al. 2002; Quicke 2015) and Sanger DNA sequence data (Sharanowski et al. 2011; Zaldívar-Riverón et al. 2006). Recent studies using genomic-scale data, including nuclear ultraconserved elements (UCEs; Jasso-Martínez et al. 2021, 2022a) and mitochondrial genome sequences (Jasso-Martínez et al. 2022b), have significantly advanced the understanding of the generic composition and phylogenetic relationships within this group. These phylogenomic analyses consistently place Rhysipolinae as sister to the Hormiinae + Rogadinae clade, incorporating the taxonomically problematic genera *Allobracon* Gahan, 1915 and *Parachremylus* Granger, 1949, which were previously classified within the subfamily Hormiinae (Hormiini) due to their unsclerotised terga (Belokobylskij 1993; Wharton 1993).

Currently, Rhysipolinae lacks exclusive external morphological features that reliably distinguish it from other braconid subfamilies, and instead it is diagnosed by a combination of plesiomorphic and partly apomorphic features (Spencer and Whitfield 1999). For instance, Rhysipolinae shares with Hormiinae, Exothecinae, and Rogadinae several features, including an occipital carina that does not join the hypostomal carina ventrally, the presence of epicnemial carina, and sometimes costate sculpture on the second metasomal tergum (Sharkey 1997; Whitfield and Wharton 1997). However, these features vary considerably among rhysipoline genera and their sister groups (van Achterberg 1995; Quicke 2015).

In this study, we employed nuclear ultraconserved element (UCE) data for nine of the ten currently recognised rhysipoline genera, representing the first phylogenetic study specifically focused on this subfamily. Using the reconstructed phylogenetic framework and external morphological features, we evaluated the validity of the examined genera and proposed corresponding taxonomic changes. Moreover, the integration of phylogenetic and morphological evidence led to the description of a new rhysipoline genus and species, *Rogapolisnomai* gen. et sp. nov., characterised by a distinctive feature mainly known in the cyclostome subfamily Rogadinae: a median longitudinal carina on the second metasomal tergum. Finally, we provide an identification key to the valid genera of Rhysipolinae.

## ﻿Materials and methods

### ﻿Taxon sampling

We analysed UCE data from 29 species representing nine of the 11 rhysipolinae genera: *Rhysipolis*, 15 spp.; *Cantharoctonus*, 1 sp.; *Pachystigmus*, 2 spp., *Pseudavga* Tobias, 1964, 2 spp., *Parachremylus*, 2 spp.; *Allobracon*, 3 spp.; *Pseudorhysipolis* Scatolini, Penteado-Dias & van Achterberg, 2002, 2 spp.; *Cerophanes* Tobias, 1971, 1 sp.; and *Troporhysipolis* Quicke, Belokobylskij & Butcher, 2023, 1 sp. As outgroups, we included 28 species belonging to 23 genera of the subfamilies Hormiinae and Rogadinae, as these groups have consistently been recovered as sisters to Rhysipolinae in previous UCE-based studies (Jasso-Martínez et al. 2021; Jasso-Martínez et al. 2022a, b). We also included one specimen with uncertain generic assignment (DNA sample voucher: USNMENT01322932).

New UCE data were generated for 24 ingroup and outgroup species, while data for the remaining species were obtained from four previously published studies (Jasso-Martínez et al. 2021, 2022a, b; Shimbori et al. 2024). Detailed information of the specimens examined in this study, their species assignment, locality, DNA voucher, and SRA accession numbers are available in the Suppl. material 1.

### ﻿Morphological examination

We examined the external morphology of the sequenced and additional specimens, all of which are deposited in the following collections: Colección Nacional de Insectos del Instituto de Biología de la Universidad Nacional Autónoma de México (CNIN IB-UNAM); Zoological Institute of the Russian Academy of Sciences, St Petersburg, Russia (ZISP); and Coleção Entomológica, Departamento de Ecologia e Biologia Evolutiva, Universidade Federal de São Carlos, São Carlos, SP, Brazil (DCBU). Morphological terminology follows van Achterberg (1988), except for wing venation and microsculpture features, which follow Sharkey and Wharton (1997) and Harris (1979), respectively. Digital images of representative species from various rhysipoline genera were taken at the Zoological Institute of the Russian Academy of Sciences using a Canon EOS 70D digital camera mounted on an Olympus SZX10 microscope, and at the Laboratorio Nacional de Biodiversidad (LANABIO) at IBUNAM using a ZEISS^®^ AXIO ZoomV16 stereoscopic microscope, an AxioCam MRc5 (5 megapixels) camera, and the ZEN 2012 (Blue Edition) software.

### ﻿DNA extraction protocol and library preparation

Genomic DNA was extracted from ethanol-preserved and pinned specimens using a non-destructive technique (Ceccarelli et al. 2012) with the EZ-10 Spin Column Genomic DNA minipreps Kit (BIOBasic, Toronto, ON, Canada). Specimens were digested overnight, and subsequently removed from digestion, washed with distiller water, and remounted. DNA quantification was performed using a Qubit 4.0 fluorometer (v.4.0, Invitrogen, Life Technologies, Carlsbad, CA, USA) and the High Sensitivity Kit (Invitrogen, Life Technologies, Carlsbad, CA, USA).

Twenty-two genomic libraries were prepared following the protocol described by Branstetter et al. (2017), using the Kapa Hyper Prep Kit (Kapa Biosystems Inc., Wilmington, MA, USA) and custom TruSeq-style dual-indexing barcodes adapters (Glenn et al. 2019) for *in silico* demultiplexing. For library preparation, up to 150 ng of input DNA per sample was resuspended in 100 μl of ultrapure water. DNA was sheared into ~ 200–600 bp fragments using a BioRuptor Pico sonicator, applying one to three cycles of 15–90-second on/off pulses, depending on the collection date and condition of each specimen. Samples were pooled at equimolar concentrations in groups of 7–10 libraries for enrichment, with a total input of 2,000 ng of DNA per enrichment reaction. The UCE enrichment was performed using the RNA probe set Hym v.2 designed for Hymenoptera (Branstetter et al. 2017), which includes 31,829 baits targeting 2,590 UCE loci, following the standard enrichment protocol (www.ultraconserved.org). Post-enrichment DNA pools were quantified using the Qubit 4.0 fluorometer with the Broad Range Kit (Invitrogen, Life Technologies, Carlsbad, CA, USA), combined at equimolar ratios, and sent for sequencing to Admera Health BioPharma Services (South Plainfield, NJ, USA) employing an Illumina NovaSeqX instrument (PE150, v4 chemistry). Sequenced libraries produced 150-bp paired-end reads.

Two additional libraries belonging to two species of *Pseudorhysipolis* were generated using the NEBNext Ultra II FS DNA Library Prep kit (New England Biolabs, Ipswich, MA, USA) according to the manufacturer’s protocol, scaled to a 1:15 ratio. Up to 10 ng of DNA, resuspended in 2.6 µL of ultrapure water, was used as input. DNA was fragmented for 5 minutes to achieve a mean fragment size of 200–500 bp. Amplified and purified libraries were quantified using Qubit, pooled at equimolar concentrations, and sent for sequencing to Admera Health BioPharma Services (South Plainfield, NJ, USA). Sequencing was performed on an Illumina NovaSeqX instrument (PE150, v4 chemistry). Sequenced libraries produced 150-bp paired-end reads. Raw sequence data for all newly generated samples are available in the NCBI Sequence Read Archive (NCBI-SRA) under BioProject accession number PRJNA1228065.

### ﻿UCE data processing

Bioinformatic processing was performed using the Beagle HPC supercomputer at the Instituto de Biología, Universidad Nacional Autónoma de México (IB-UNAM). Raw reads were cleaned of adapters, and low-quality sequences were filtered using Trimmomatic v. 0.39 (Bolger et al. 2014). Reads assembly was performed on the web server Galaxy (usegalaxy.org) using either SPAdes or RNAspades (Bankevich et al. 2012; Bushmanova et al. 2019). Contigs were processed following the Phyluce v. 1.7.1 pipeline (Faircloth 2016).

UCE contigs were identified using the Hymenoptera-v2 probe set (Branstetter et al. 2017) and subsequently extracted. Individual UCE loci were aligned using MAFFT (Katoh and Toh 2008) implemented in Phyluce, and poorly aligned regions were removed using GBLOCKS v. 0.91 (Talavera and Castresana 2007) with relaxed stringency values (0.5, 0.5, 12, and 7 for the b1-b4 parameters, respectively). This bioinformatic pipeline was applied to both target and non-target enrichment samples. Finally, we built completeness matrices with thresholds of 50, 60, 70%.

### ﻿Phylogenetic analyses

We used the SWSC-EN algorithm (Tagliacollo and Lanfear 2018) to define partitions within each UCE locus. The optimal partition scheme and appropriate evolutionary model for each partition was determined using ModelFinder in IQTREE v2.2.0 (Minh et al. 2020) with the -rclusterf option, which is suitable for our data matrices (Lanfear et al. 2017) and the -TESTMERGEONLY command, which implements the greedy algorithm of PartitionFinder. We also used the Bayesian information criterion (BIC) to identify the best partition scheme. Maximum likelihood (ML) analyses were conducted using IQTREE v. 2.2.0 (Minh et al. 2020) with 1,000 ultra-fast bootstrap replicates (Hoang et al. 2018) to assess clade support and generate a consensus tree (Soltis and Soltis 2003).

All completeness matrices with their respective partition schemes and their derived phylogenetic trees are available in the FigShare repository (10.6084/m9.figshare.28489664).

## ﻿Results

### ﻿UCE performance and alignment statistics

An average of 1,618,742 reads were obtained for the newly processed samples prior to filtering and trimming. After trimming, an average of 1,290,498 clean reads were obtained. The cleaned reads produced an average of 95,360 assembled contigs (min. 33,351 – max. 470,913). We recovered a total of 2,460 UCE loci from the 2,590 available loci in the Hymenoptera-v2 probe set across all samples, including outgroups. The recovered loci had a mean length of 333.60 bp after aligning and trimming. The average number of UCE loci recovered was 1,192.1. *Parachremylus* sp. and *Sorayaalencarae* had the lowest (99) and highest (1898) number of loci, respectively, across all samples. The alignment summary for each completeness matrix is provided in Table 1.

**Table 1.** Alignments summary for the matrices with different completeness percentages.

Matrix	No. of taxa	UCE loci	Loci mean length (bp) [min-max]	Matrix length (bp)	Informative sites	Nucleotide positions
50%	61	1,232	371.07 [172-614]	457,160	218,966	15,871,303
60%	61	827	379.36 [200-614]	313,727	160,106	12,249,152
70%	61	642	388.45 [200-614]	249,388	132,562	10,357,365

### ﻿Phylogenetic relationships

**Figure 1.**
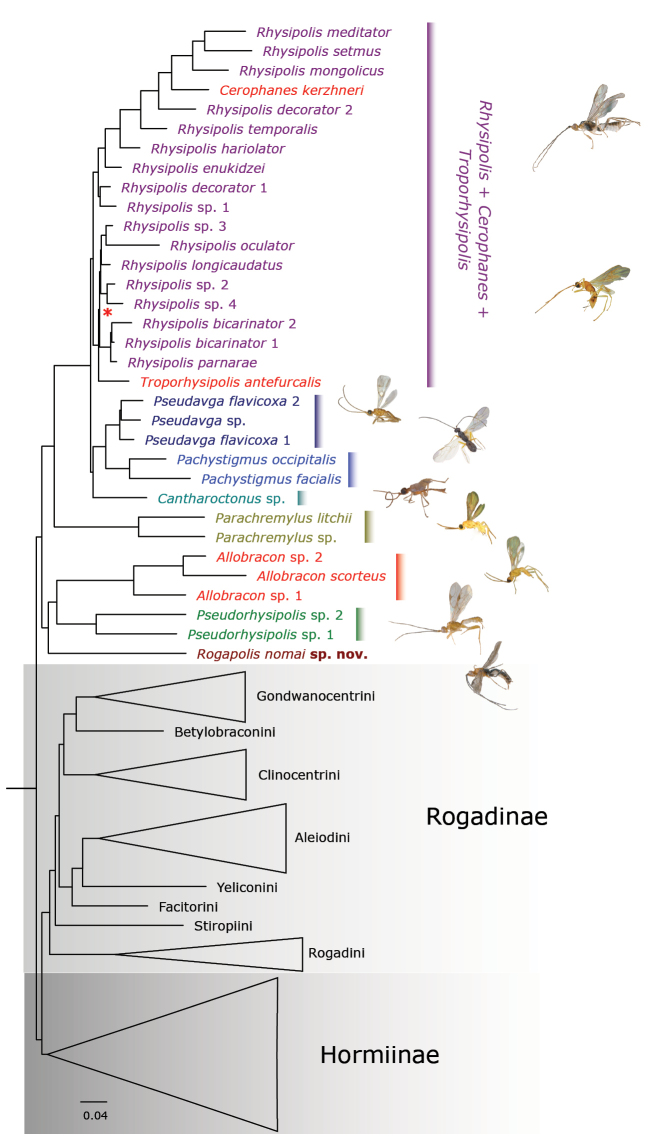
ML phylogram derived from the 50% completeness matrix. Coloured clades correspond to the different rhysipoline genera, except for the *Rhysipolis* (violet), *Cerophanes* (red) and *Troporhysipolis* (red) clade. Asterisks (*) near branches indicate bootstrap support values *<* 100. Nodes without labels are supported by BTP support values of 100.

The ML phylograms derived from the analyses conducted with the 50%, 60% and 70% completeness matrices are provided in Fig. 1 and Suppl. material 2. We recovered similar topologies for the analyses across the three completeness matrices, with topological differences primarily observed among species of the *Rhysipolis* + *Cerophanes* + *Troporhysipolis* clade. The topology from the 50% completeness matrix had the highest number of nodes supported by bootstrap (BTP) values of 100 (all but one node within the ingroup), and we thus only describe its relationships.

The subfamily Rhysipolinae and most of its genera were recovered as monophyletic, except for *Rhysipolis*, which was paraphyletic with respect to the only species of *Cerophanes* and *Troporhysipolis*: *C.kerzhneri* Tobias and *T.antefurcalis* (Granger). The *Rhysipolis* clade was divided into two main subclades. The first included *C.kerzhneri* deeply nested along with most *Rhysipolis* species from the Palaearctic and one from the Nearctic regions. The second subclade comprised *Rhysipolis* species from the Palaearctic, Oriental, and Neotropical regions, with the Afrotropical *T.antefurcalis* placed as sister to all of them.

The species with problematic assignment was recovered as sister to the *Pseudorhysipolis* + *Allobracon* clade, with these three taxa being sister to the remaining rhysipoline genera. *Parachremylus* was also recovered as monophyletic and sister to the *Cantharoctonus* + (*Pseudavga* + *Pachystigmus*) and the *Rhysipolis* + *Cerophanes* + *Troporhysipolis* clades. *Cantharoctonus*, on the other hand, was sister to the *Pseudavga* + *Pachystigmus* clade.

### ﻿Taxonomic inferences

Our taxon sampling confirmed the monophyly of Rhysipolinae and of most of its genera. However, *Rhysipolis* was recovered as paraphyletic with respect to *Cerophanes* Tobias and *Troporhysipolis*. These two genera are morphologically similar to *Rhysipolis*, sharing all the diagnostic features of this genus, including a complete occipital carina not joining the hypostomal carina, epicnemial carina complete, and first to third terga never striated. *Cerophanes* is distinguished from *Rhysipolis* by the presence of a large inner horn-like process on the scapus (absent in *Rhysipolis*) (Tobias 1971; Belokobylskij and Tobias 1986; Whitfield and Wagner 1991), whereas *Troporhysipolis* is distinguished by the vein 1cu-a antefurcal to the veins 1M and 2CUb arising distinctly before the middle of distal margin of second subdiscal cell (vein 1cu-a postfurcal to vein 1M and vein 2CUb usually arrising behind or in the middle of distal margin of second subdiscal cell in *Rhysipolis*) (Quicke et al. 2016). Our best-supported phylogenetic estimate placed *Cerophanes* deeply nested within *Rhysipolis*, whereas *Troporhysipolis* appeared at the base of one of the two main subclades of *Rhysipolis*. Based on these relationships and on the aforementioned morphological features, we propose *Cerophanes* syn. nov. as a junior synonym of *Rhysipolis*. Further molecular phylogenetic studies including additional species of *Troporhysipolis* are needed to confirm its generic status.

Our best phylogenetic estimate recovered the members of *Pachystigmus* and *Pseudavga* as reciprocally monophyletic, which is congruent with their morphology. Species of both genera share several features, including a short first metasomal tergum, typically not longer than its posterior width, basal sternal plate not longer than its posterior width, vein CU1a of fore wing arising from middle of distal margin of second subdiscal cell, and vein m-cu of hind wing usually present. Tobias (1964) described *Pseudavga* and distinguishing it from *Avga* Nixon, 1940 and *Hormius* Nees, 1919, though overlooked the genus *Noserus* Foerster, 1863 (= *Pachystigmus* Hellén, 1927), whose status was unclear at that time. A subsequent study of the type material and additional specimens of the genotype *Noserusfacialis* Förster, 1863 (Belokobylskij and Tobias 1986) revealed that this genus and *Pseudavga* did not have external morphological differences, and thus the former was regarded a synonym of the second. Recently, the restoration of the generic status of *Pseudavga* was proposed after studying its biology and subtle morphological features (Shaw and Sims 2015). However, based on our recovered relationships, molecular evidence, and on the consistent morphological similarity of these two taxa, we propose *Pseudavga* syn. nov. to be treated as a synonym of *Pachystigmus* and consider it a subgenus within the latter.

Based on the recovered phylogenetic relationships and the morphological examination of the studied specimens, we propose that the specimen with problematic assignment, which is sister to *Allobracon* + *Pseudorhysipolis*, represents an undescribed genus and species. Below, we describe this genus, provide a diagnosis for Rhysipolinae, and a key to the currently valid genera of this subfamily. Digital pictures of representative species belonging to all the rhysipoline genera recognised in this study except *Rogapolis* gen. nov. are provided in the Suppl. material 3.

### ﻿Systematic accounts

**Family Braconidae Nees, 1811**

#### ﻿Subfamily Rhysipolinae

Taxon classificationAnimaliaHymenopteraBraconidae

Belokobylskij, 1984

1EC94555-B3D5-5D56-9E40-93873A22F846

##### Diagnosis.

Head with subcircular or weakly oval hypoclypeal cavity; occipital carina usually present (except *Allobracon*), complete, not joining hypostomal carina ventrally, distinctly removed from it and separately reaching lower margin of head capsule near mandible, or sometimes incomplete ventrally; postgenal bridge always absent; maxillary palpus 6-segmented, labial palpus 4-segmented, third segment of labial palpus never shortened. Antenna often setiform, sometimes curled into ring apically in dried specimens; first flagellar segment not shorter than second segment. Mesosoma: notauli on mesoscutum complete or often absent in posterior half of mesoscutum, usually without longitudinal furrow medio-posteriorly; prepectal carina and precoxal sulcus present and distinct, but sometimes some of these structures absent (*Allobracon* and *Parachremylus*); propodeum often without areola, but sometimes with mid-longitudinal carina or with relatively several distinct areas (at least posteriorly) delineated by rather distinct carinae. Fore wing with marginal cell always closed distally, usually not shortened and reaching wing apex. Vein m-cu usually antefurcal to vein 2-SR; veins 2SR and r-m present; discal cell petiolate anteriorly; second subbasal cell always closed distally by vein CU1b; vein CU1a never interstitial; vein a always absent. Hind wing with three hamuli; vein m-cu usually present, but sometimes short, or absent; vein cu-a always present and closing subbasal cell. Subbasal cell medium-sized or short. Fore tibia without spines; hind coxa suboval, without basoventral corner and tubercle; hind femur long and narrow; claws simple and small. First metasomal tergum always with dorsope, though sometimes small; acrosternite (basal sternal plate) of first segment predominantly short, rarely (*Cantharoctonus*) elongated; dorsal carinae usually distinct at least in basal half, fused or not fused subbasally. Following terga usually relatively soft, mainly smooth, but sometimes second and third terga rather distinctly sclerotised, shagreened, granulate or even partly striate (*Afrorhysipolis*, *Rogapolis* and *Pseudorhysipolis*); laterotergites of second tergum often not separated, but usually with inflection and crease; spiracles of second and third terga situated dorso-laterally, slightly above crease; suture between second and third terga usually present, distinct, or almost indistinct. Ovipositor short, distinctly shorter than metasoma, usually slightly widened subapically, with dorsal node, often without serration ventro-apically.

##### Included genera.

*Afrorhysipolis*, *Allobracon*, *Cantharoctonus*, *Pachystigmus* (= *Pseudavga*, *Noserus*), *Parachremylus*, *Pseudorhysipolis*, *Rhysipolis* (= *Cerophanes*), *Rogapolis*, *Troporhysipolis* (Table 2).

**Table 2.** List of valid rhysipoline genera after this study, including author, geographic distribution, and number of their described species (Yu et al. 2016; Jasso-Martínez et al. 2021, 2022a, b).

Genera	Author and year	Geographic distribution	No. described species
*Afrorhysipolis*	Belokobylskij, 1999	Afrotropical	1
*Allobracon*	Gahan, 1915	Nearctic, Neotropical	24
*Cantharoctonus*	Viereck, 1912	Nearctic, Neotropical	9
*Pachystigmus* (*Pseudavga* syn. nov.)	Hellén, 1927	Afrotropical, Palaearctic	6
*Parachremylus*	Granger, 1949	Afrotropical, Oriental	4
*Pseudorhysipolis*	Scatolini, Penteado-Dias & van Achterberg, 2002	Neotropical	10
*Rhysipolis* (*Cerophanes* syn. nov.)	Förster, 1863	Neotropical, Nearctic, Oriental, Palaearctic	24
*Rogapolis*	gen. nov.	Neotropical	
*Troporhysipolis*	Quicke, Belokobylskij & Butcher, 2016	Afrotropical, Australasian	4

##### Comments.

The genus *Neoavga* Belokobylskij, 1989 was originally included within Hormiinae (Hormiini; Belokobylskij 1989) and it was subsequently transferred to Rhysipolinae (van Achterberg 1995; Yu et al. 2016), though Wharton (1993) previously suggested that this genus belonged to Exothecini. More recently, in the redefinition of the Mesostoinae (Shimbori et al. 2017), *Neoavga* was proposed to belong to this subfamily mainly based on the presence of the crossvein a in the fore wing, and the epicnemial carina present only laterally and absent ventrally. This placement was later confirmed by Quicke et al. (2020) in a molecular phylogenetic study that focused on the cyclostome braconid subfamilies.

#### ﻿ Rogapolis

Taxon classificationAnimaliaHymenopteraBraconidae

García-Acosta, Shimbori, Castañeda-Osorio & Zaldívar-Riverón gen. nov.

7241A672-7FE7-5362-9090-890A444C7CFE

https://zoobank.org/499133C5-4432-4361-B0FE-1296986DCA14

##### Type species.

*Rogapolisnomai* sp. nov.

##### Diagnosis.

*Rogapolis* can be morphologically distinguished from the remaining members of Rhysipolinae by having the second metasomal terga with a basal triangular median area followed by a longitudinal carina, a feature that had been mainly observed in most members of the subfamily Rogadinae and some Braconinae.

##### Description.

***Head***: Antenna with at least 45 flagellomeres. Basal flagellomeres long, distal flagellomeres shorter. Distal margin of scapus strongly oblique (ventral length of pedicellus as long as ventral length of scapus). Frons, vertex, temple, and gena smooth and polished. Eyes glabrous, large, and oval-shaped. Malar space relatively short, distinctly shorter than eye. Face considerably pilose, with long setae. Hypoclypeal depression small and rounded. Malar suture present. Frons depressed, flat, with an indistinct median transversal carina. Ocelli small. Occipital carina incomplete medio-dorsally, ventrally not joining hypostomal carina.

***Mesosoma***: Mostly smooth and polished, except metapleuron and propodeum, which are rugose areolate. Propleuron with posterior flange. Notauli deep, wide, not joining posteriorly, finishing in the middle of mesoscutum. Mid pit absent. Scutellar sulcus with six complete carinae. Epicnemial carina present. Precoxal sulcus deep, scrobiculate, extended at least two thirds length of mesopleuron. Metanotum with complete mid-longitudinal carina, posterior margin not protruding. Propodeum angled in lateral view, with median longitudinal carina present.

***Wings***: Forewing vein r as long as vein (RS+M)a, inserted in the proximal part of the pterostigma, slightly oblique; second submarginal cell moderately large, rectangular, distinctly narrowing proximally, vein r-m present but spectral; vein 1RS short; vein (RS+M)a slightly sinuate; vein M+CU completely tubular and almost straight; 1cu-a postfurcal; vein 2cu-a present and long. Hindwing veins RS and M present; vein M+CU as long as vein 1-M; second subdiscal cell long and closed distally; vein m-cu present and distinctly sclerotised.

***Legs***: Coxae mostly smooth, with long setae. Hind tibial spurs slightly curved and with few setae. Claws simple, without distinct basal lobe or pecten.

***Metasoma***: First and second terga longitudinally costate; first tergum with a median longitudinal carina; second tergum with a basal triangular median area followed by median longitudinal carina. Exposed part of ovipositor sheath short, 0.5× as long as hind tibia.

##### Biology.

Unknown.

##### Geographic distribution.

This genus is known only from the type locality, a cloud forest region in the state of Acre, northern Brazil.

##### Etymology.

The genus name *Rogapolis* is formed by combining Rogadinae, a subfamily that includes morphologically similar genera, and *Rhysipolis*, a genus within Rhysipolinae, the subfamily to which this new genus belongs. The gender of the genus is feminine, following the grammatical treatment of taxonomic names ending in -*polis*.

#### ﻿ Rogapolis nomai

Taxon classificationAnimaliaHymenopteraBraconidae

García-Acosta, Shimbori, Castañeda-Osorio & Zaldívar-Riverón sp. nov.

6EFF9E2B-6594-5B11-9ABF-26892AEB7CA1

https://zoobank.org/9AC078D0-A04E-4004-B2B1-39CF12AD842C

Fig. 2A–I

##### Type material.

***Holotype***, female (USNMENT01322932) “BRAZIL, Mâncio Lima, AC / 20.IV.2006 / Menezes col” (DCBU 22093).

**Figure 2.**
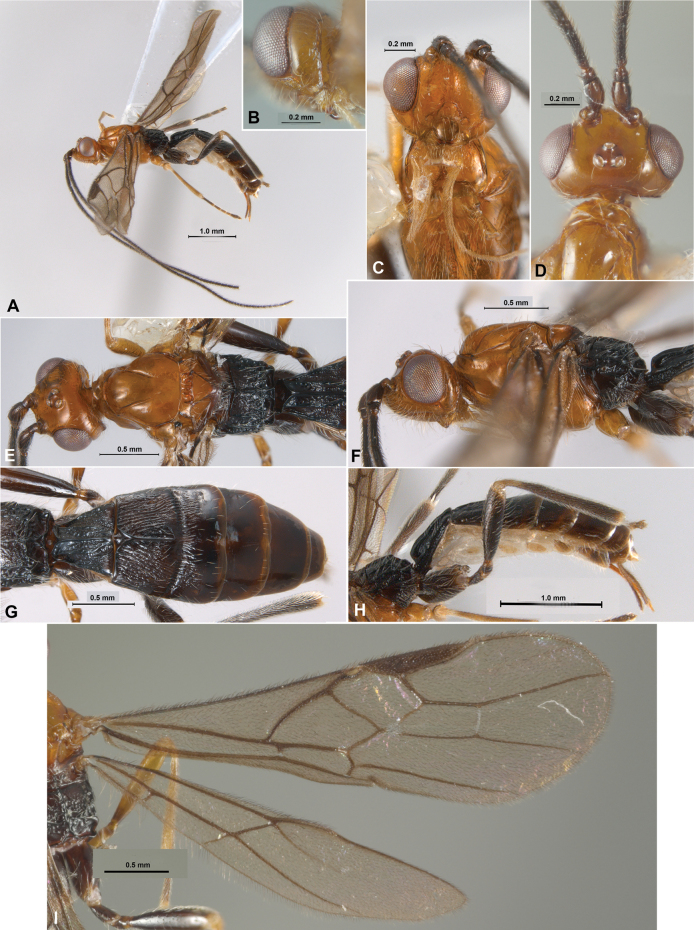
*Rogapolisnomai* García-Acosta, Shimbori, Castañeda-Osorio & Zaldívar-Riverón, gen. et sp. nov. Holotype, female **A** habitus, lateral view **B** head, posterolateral view (shown hypostomal carina not reaching occipital carina) **C** head, front view **D** head, dorsal view **E** head and mesosoma, dorsal view **F** head and mesosoma, lateral view **G** metasoma, dorsal view **H** metasoma, lateral view **I** wings.

##### Description.

Female, body length 4 mm, fore wing 4 mm; antenna 6.1 mm.

***Head*.** Face without mid-longitudinal ridge, smooth and polished. Frons, occiput, temples, and malar space smooth and polished. Frons depressed behind antennal sockets, flat, with a an almost indistinct median transversal carina. Occipital carina medio-dorsally incomplete, ventrally present, and not joining hypostomal carina above the base of mandible. Temple narrow and receding (dorsal view), about as long as eyes. Head in dorsal view 1.25× wider than mesoscutum height. POL 0.57× OD, 0.238× OOL. Face width 1.51× longer than hight. Hypoclypeal cavity nearly round. Diameter of hypoclypeal cavity 0.69× shorter than distance between cavity and eye margin. Hypoclypeal cavity moderate to strongly convex dorsally. Antenna 1.5× longer than body. Flagellum with 45 flagellomeres (one broken). Apical (lighter) flagellomeres somewhat widening in comparison with basal ones. First flagellomere 3.4× longer than wide, 1.55× longer than the second and 1.65× third.

***Mesosoma*.** Pronotal collar forming a distinct neck. Pronotum smooth and polished. Pronope and subpronopes present. Pronope forming a wide concave area. Mesosoma 1.79× longer than high, 2.2× longer than width in dorsal view. Mesoscutum and scutellum smooth and polished. Mesoscutum 0.97× longer than high. Scutellum 1.9× wider than long. Scutellar sulcus 0.4× as long as wide.

***Wings*.** Forewing: pterostigma relatively small, 5.4× longer than maximum width. Vein 2RS 1.4× longer than vein r. Vein 3RSa 1.17× longer than 2RS. Vein 3RSb 2.3× longer than 3RSa; vein 1CUa around same size than vein 1cu-a; vein (RS+M)a straight. Hind wing: vein 1M 1.31× longer than M+CU. Vein 1M 3.2× longer than m-cu.

***Legs*.** Mid and hind coxae smooth and polished. Hind legs with tarsi broken. Ventral margin of hind tibia without dense comb of setae. Hind tibial spurs slightly curved with few setae.

***Metasoma*.** First metasomal tergum short, 2.1× longer than subbasal width, 0.8× as long as distal width. First and second terga longitudinally costate. Third tergum longitudinally costate anteriorly. Ovipositor sheaths uniformly setose, short, 0.5× as long as hind tibia.

***Colour*.** Head, mesoscutum, scutellum, and mesopleuron honey yellow, propodeum and metasoma dark brown to black. Antenna, fore and mid coxae, and tibiae honey yellow. Hind leg and fore and middle tarsi dark brown to black. Wings dusky; veins and pterostigma brown to dark brown. Ovipositor sheaths dark brown. Ovipositor honey yellow.

**Male.** Unknown.

##### Etymology.

This species was named after a fictional alien race called Nomai, from the ‘Outer Wilds’ video game.

### ﻿Key to Rhysipolinae genera

**Table:** 

1	Prepectal carina present and complete. Occipital carina usually complete, sometimes reduced ventrally. First metasomal tergum entirely coarsely sclerotised, without membranous areas in posterior half. Mesoscutum usually mostly smooth	**2**
–	Prepectal carina absent. Occipital carina incomplete, reduced dorsally and ventrally, or sometimes entirely absent. First metasomal tergum with two large, membranous areas in posterior half. Mesoscutum densely finely granulate	**8**
2	Vein m-cu of fore wing distinctly postfurcal to vein 2-SR. Mesoscutum densely granulate. Second and third metasomal terga distinctly sclerotised and mainly rugose-striate. Afrotropical region	***Afrorhysipolis* Belokobylskij, 1999**
–	Vein m-cu of fore wing distinctly antefurcal to vein 2-SR. Other characters variable	**3**
3	First and second metasomal terga with posteriorly acuminate subtriangular basal areas and with high medial carinae prolonged to posterior margins of terga. First and second metasomal terga entirely and third tergum in basal half distinctly striate with reticulation between striae. Neotropical region	***Rogapolis* gen. nov.**
–	First and second metasomal terga usually without acuminate subtriangular basal areas and usually without high medial carinae prolonged to posterior margins of terga. First to third metasomal terga never striate, usually smooth and often weakly sclerotised, but sometimes shagreened or granulate (some *Pseudorhysipolis* species)	**4**
4	Vein cu-a of fore wing distinctly antefurcal to vein 1-M. Second subdiscal cell of fore wing concave, widened medially. Vein 2-CU1 thickened. Vein CU1 of fore wing arising before or from middle of distal margin of second subdiscal cell. Afrotropical region	***Troporhysipolis* Quicke, Belokobylskij & Butcher, 2016**
–	Vein cu-a of fore wing distinctly postfurcal to vein 1-M (basal). Second subdiscal (brachial) cell of fore wing not widened medially. Vein 2-CU1 not thickened. Vein CU1 of fore wing variable	**5**
5	Inner apex of hind tibia with conspicuous comb of setae. Vertex, mesoscutum and propodeum antero-dorsally granulate. Vein 1-M of hind wing 1.5–2.2× as long as vein M+CU. Second metasomal terga often entirely and most part of third tergum strongly sclerotised and densely granulate. Neotropical region	***Pseudorhysipolis* Scatolini, Penteado-Dias & van Achterberg, 2002**
–	Inner apex of hind tibia without comb or with a comparatively narrow comb of setae. Vertex, mesoscutum and propodeum without granulation. Vein 1-M of hind wing 1.3× as long as vein M+CU or less. Second and third terga always weakly sclerotised and smooth or faintly shagreened	**6**
6	Setose part of ovipositor sheath longer than half of metasoma. Second subdiscal cell of fore wing wide. Vein CU1a of fore wing arising from posterior 0.3 of distal margin of second subdiscal cell. Occipital carina present and complete, not fused below with hypostomal carina, removed from it and separately reaching lower margin of head capsule near mandible. Vein r of fore wing arising near middle of pterostigma. Rarely scapus of antenna modified, with distinct and acuminate inner lateral process. Nearctic, Neotropical, Oriental, and Palaearctic regions	***Rhysipolis* Foerster, 1863** [*Cerophanes* Tobias, 1960, syn. nov.]
–	Setose part of ovipositor sheath short or very short, not or only slightly longer than first metasomal tergum. Second subdiscal cell of fore wing narrow. Vein CU1a arising from or before middle of distal margin of second subdiscal cell. Occipital carina present, fused with hypostomal carina, or not fused, removed from it and separately reaching lower margin of head capsule near mandible. Vein r of fore wing usually arising distal to middle of pterostigma, often from its distal 0.3–0.4. Scapus never modified	**7**
7	Propodeum with wide, transverse groove anteriorly. First metasomal tergum long, 1.5–1.8× longer than its posterior width; basal sternal plate elongated. Vein CU1a of fore wing often arising before middle of distal margin of second subdiscal cell. Vein m-cu of hind wing absent. Pterostigma of male never enlarged. Nearctic and Neotropical regions	***Cantharoctonus* Viereck, 1912**
–	Propodeum usually without transverse groove anteriorly, sometimes only with narrow sulcus. First metasomal tergum short, usually not longer than its posterior width; basal sternal plate never elongated. Vein CU1a of fore wing always arising from middle of distal margin of second subdiscal cell. Vein m-cu of hind wing usually present. Pterostigma of male sometimes enlarged. Afrotropical and Palaearctic regions	***Pachystigmus* Hellen, 1927** [including *Pseudavga* Tobias, 1964 as subgenus]
8	Occipital carina completely absent. Vertex smooth. Precoxal sulcus absent. Propodeum without areola, with distinct and almost complete longitudinal keel. Second and third metasomal terga without longitudinal carina, rarely second tergum basally with short carina. Nearctic and Neotropical regions	***Allobracon* Gahan, 1915**
–	Occipital carina present laterally, absent dorsally and sometimes ventrally. Vertex densely granulate-coriaceous. Precoxal sulcus present. Propodeum with large areola delineated by distinct carinae, longitudinal carina absent or, if present, short basally. Second and third metasomal terga with distinct median longitudinal carina. Afrotropical and Oriental regions	***Parachremylus* Granger, 1949**

## ﻿Discussion

This study represents the first effort to delineate the limits of the subfamily Rhysipolinae using nuclear genome-wide data. As a small and historically overlooked braconid subfamily with a global distribution, our findings also provide a phylogenetic framework to assess the validity of its genera. Our best phylogenetic estimate consistently supports the monophyly of the subfamily with the inclusion of the newly described genus *Rogapolis*. However, our morphological revision failed to identify any diagnostic morphological feature exclusive to Rhysipolinae, leaving its monophyly currently supported solely by molecular data.

A putative synapomorphy of Rhysipolinae is their biology as koinobiont ectoparasitoids of leaf-miners and leaf-rollers. However, evidence supporting this trait is scarce and limited to only two of the nine genera (Shaw 1983; Shaw and Sims 2015; Shaw 2017). While host associations are documented for *Allobracon*, *Parachremylus*, and *Troporhysipolis*, the available information is incomplete. *Allobracon* is associated with leaf-miners, but its parasitism strategy remains unknown (Muesebeck 1958; Belokobylskij and Maeto 2006; Quicke et al. 2016). Similarly, *Troporhysipolis* has hosts among leaf-tying Lepidoptera rather than leaf-miners, and no further details on its biology are available (Quicke et al. 2016). A second putative synapomorphy of this subfamily is the presence of large abdominal exocrine glands in males, a feature originally described by Buckingham and Sharkey (1988) in *Rhysipolis* (though not examined in other rhysipoline genera). These glands are morphologically similar to the so-called Hagen’s glands observed in the subfamilies Opiinae, Alysiinae, and Telengaiinae (formerly Gnamptodontinae), and thus their presence was interpreted as indicative of a phylogenetic affinity between Rhysipolinae and the latter three subfamilies (Buckingham and Sharkey 1988). However, this hypothesis has been refuted based on recent molecular-phylogenetic evidence (Jasso-Martínez et al. 2022a, b). Notably, Buckingham and Sharkey (1988) highlighted key anatomical differences, particularly in the position of the gland openings, underscoring their morphological significance. Given the potential phylogenetic relevance of this structure, further investigation into its prevalence within Rhysipolinae could provide additional insights into the evolutionary history of this group.

Despite the absence of known exclusive diagnostic morphological features, a combination of previously proposed characters can help distinguish Rhysipolinae from its closely related subfamilies Hormiinae, Exothecinae and Rogadinae (Belokobylskij 1993; Wharton 1993; van Achterberg 1995). These characters include the occipital carina not merging with hypostomal carina, complete epicnemial carina, vein m-cu of fore wing usually antefurcal to vein 2-SR, vein cu-a of fore wing usually postfurcal to vein 1-M, submedian vein of forewing not strongly decurved, and a weakly developed forewing SR.

Following the taxonomic changes proposed in this study, we recognise nine genera within Rhysipolinae (Table 2), several of which possess distinctive diagnostic features. For example, *Rogapolis* is mainly defined by a median longitudinal carina on the second metasomal tergum, a feature that is present in most rogadine genera (Sharkey 1997), but that within Rhysipolinae is only shared with *Parachremylus*. *Pseudorhysipolis*, on the other hand, is distinguished by a conspicuous comb of modified bristles on the hind tibia and an often granulate sculpture of mesosoma (Scatolini et al. 2002), while *Allobracon* is characterised by the complete absence of an occipital carina and a partially unsclerotised first metasomal tergum (as in *Parachremylus*) (Belokobylskij 1993; Wharton 1993).

Our phylogenetic reconstruction revealed geographic patterns in the distribution of some clades within Rhysipolinae. Two main clades can be distinguished within the subfamily. The first only includes genera with a New World (Neotropical and Nearctic) distribution—*Rogapolis*, *Pseudorhysipolis*, and *Allobracon*—, whereas the second consists of genera primarily distributed across the Afrotropical, Oriental, and Palaearctic regions—*Pachystigmus*, *Parachremylus*, *Rhysipolis*, and *Troporhysipolis*— though it also contains the Neotropical and Nearctic genus *Cantharoctonus*.

The fossil subgenus of *Rhysipolis*, Rhysipolis (Granulopolis) simutniki Belokobylskij, 2024, was recently described from inclusion of the late Eocene Baltic amber, revealing that this genus was already extant during the late Eocene, 37-34 Mya (Belokobylskij and Manukyan 2024). All previous records of rhysipoline (in current sense) fossil taxa from the Baltic amber, amber from the Tarkeshwar lignite mine in Gujarat, India, and imprints of Rott am Siebengebirge. Mount and Aix-en-Provence in France, are unconfirmed and therefore doubtful (Belokobylskij and Manukyan 2024). Further UCE-based phylogenomic studies incorporating more rhysipoline species from different continents are therefore necessary to evaluate the origin and subsequent diversification of Rhysipolinae across the New and Old Worlds.

## Supplementary Material

XML Treatment for Rhysipolinae

XML Treatment for Rogapolis

XML Treatment for Rogapolis nomai

## ﻿Additional information

### Author ORCIDs

Rubén Castañeda-Osorio https://orcid.org/0000-0003-0507-5477

Sergey A. Belokobylskij https://orcid.org/0000-0002-3646-3459

Eduardo M. Shimbori https://orcid.org/0000-0003-4655-2591

Jovana M. Jasso-Martínez https://orcid.org/0000-0001-6497-7150

Angélica M. Penteado-Dias https://orcid.org/0000-0002-8371-5591

Alejandro Zaldívar-Riverón https://orcid.org/0000-0001-5837-1929

### Data availability

All of the data that support the findings of this study are available in the main text or Supplementary Information.

## Supplementary materials

Supplementary material 1 Species assignment, locality, DNA voucher number, and GenBank and SRA accession numbers of the specimens examined in this study Data type xlsx https://binary.pensoft.net/file/1302384This dataset is made available under the Open Database License (http://opendatacommons.org/licenses/odbl/1.0/). The Open Database License (ODbL) is a license agreement intended to allow users to freely share, modify, and use this Dataset while maintaining this same freedom for others, provided that the original source and author(s) are credited.Gerardo Y. García-Acosta, Rubén Castañeda-Osorio, Sergey A. Belokobylskij, Eduardo M. Shimbori, Jovana M. Jasso-Martínez, Angélica M. Penteado-Dias, Alejandro Zaldívar-Riverón

Supplementary material 2 ML phylograms derived from the 60% and 70% completeness matrices (1 and 2, respectively) Data type pdf Explanation note Numbers near branches indicate bootstrap support values. Nodes without labels are supported by BTP support values of 100. https://binary.pensoft.net/file/1302385This dataset is made available under the Open Database License (http://opendatacommons.org/licenses/odbl/1.0/). The Open Database License (ODbL) is a license agreement intended to allow users to freely share, modify, and use this Dataset while maintaining this same freedom for others, provided that the original source and author(s) are credited.Gerardo Y. García-Acosta, Rubén Castañeda-Osorio, Sergey A. Belokobylskij, Eduardo M. Shimbori, Jovana M. Jasso-Martínez, Angélica M. Penteado-Dias, Alejandro Zaldívar-Riverón

Supplementary material 3 Digital pictures of representative species belonging to all the rhysipoline genera recognised in this study except *Rogapolis* Data type pdf Explanation note **fig. 1.***Afrorhysipolisintermedius* Belokobylskij, 1999. Female, paratype. **A** habitus, lateral view **B** face **C** head, dorsal view **D** antenna, apical segments **E** antenna, scapus and pedicel **F** head and mesosoma, lateral view **G** mesosoma, dorsal view **H** metasoma, dorsal view **I** metasoma, lateral view **J** fore and hind wings **K** legs. **fig. 2.***Allobraconprimus* (Muesebeck, 1958). Female, paratype. **A** habitus, lateral view **B** face **C** head, dorsal view **D** antenna, scapus and pedicel **E** head and mesosoma, lateral view **F** mesosoma, dorsal view **G** metasoma, dorsal view **H** metasoma, lateral view **I** fore and hind wings **J** hind legs. **fig. 3.***Cantharoctonusstramineus* Viereck, 1912. Female, paratype. **A** habitus, dorsal view **B** face **C** head, dorsal view **D** antenna, scapus and pedicel **F** head and mesosoma, lateral view **G** mesosoma, dorsal view **H** metasoma, dorsal view **I** metasoma, ventral view **J** fore and hind wings **K** hind leg. **fig. 4.***Pachystigmusfacialis* (Förster, 1863). Female. **A** habitus, dorsal view **B** face **C** head, dorsal view **D** antenna, apical segments **E** antenna, scapus and pedicel **F** head and mesosoma, lateral view **G** mesosoma, dorsal view **H** propodeum and metasoma, dorsal view **I** metasoma, lateral view **J** legs **K** fore and hind wings **L** part of male fore wing. **fig. 5.***Pseudavgaflavicoxa* Tobias, 1964. Female. **A** habitus **B** face **C** head, dorsal view **D** antenna, apical segments **E** antenna, scapus and pedicel **F** mesosoma, lateral view **G** mesosoma, dorsal view **H** propodeum and metasoma, dorsal view **I** metasoma, lateral view **J** fore and hind wings **K** hind coxa and femur L hind tibia. **fig. 6.***Parachremyluslitchii* Belokobylskij & Maeto, 2006. Female. **A** habitus, lateral view **B** face **C** head, dorsal view **D** antenna, scapus and pedicel **E** antenna, apical segments **F** mesosoma, lateral view **G** mesosoma dorsal view **H** propodeum and metasoma, dorsal view **I** metasoma, lateral view **J** fore wing **K** hind wing **L** hind legs. **fig. 7.**Pseudorhysipolis (Pararhysipolis) sp. Female. **A** habitus, lateral view **B** head, dorsal view **C** face **D** head and mesosoma, dorsal view **E** head and mesosoma, lateral view **F** propodeum and metasoma, dorsal view **G** metasoma, lateral view **H** fore and hind wings. **fig. 8.**Pseudorhysipolis (Pseudorhysipolis) sp. Female. **A** habitus, lateral view **B** head, dorsal view **C** face **D** head and mesosoma, dorsal view **E** head and mesosoma, lateral view **F** propodeum and metasoma, dorsal view **G** metasoma, lateral view **H** fore wing **I** hind wing. **fig. 9.***Rhysipolismeditator* (Haliday, 1836). Female. **A** habitus, lateral view **B** face **C** head, dorsal view **D** antenna, scapus and pedicel **E** head and mesosoma, lateral view **F** mesosoma, dorsal view **G** metasoma, dorsal view **H** metasoma, lateral view **I** fore and hind wings **J** legs. **fig. 10.***Troporhysipolisantefurcalis* (Granger, 1949). Female. **A** habitus, lateral view **B** face **C** head, dorsal view **D** antenna, scapus and pedicel **E** mesosoma, dorsal view **F** mesosoma, lateral view **G** propodeum and metasoma, dorsal view **H** metasoma, lateral view **I** fore and hind wings **J** first metasomal tergum. https://binary.pensoft.net/file/1302386This dataset is made available under the Open Database License (http://opendatacommons.org/licenses/odbl/1.0/). The Open Database License (ODbL) is a license agreement intended to allow users to freely share, modify, and use this Dataset while maintaining this same freedom for others, provided that the original source and author(s) are credited.Gerardo Y. García-Acosta, Rubén Castañeda-Osorio, Sergey A. Belokobylskij, Eduardo M. Shimbori, Jovana M. Jasso-Martínez, Angélica M. Penteado-Dias, Alejandro Zaldívar-Riverón

## References

[B1] Bankevich A Nurk S Antipov D Gurevich AA Dvorkin M Kulikov AS Lesin VM Nikolenko SI Pham S Prjibelski AD Pyshkin AV Sirotkin AV Vyahhi N Tesler G Alekseyev MA Pevzner PA (2012) SPAdes: a new genome assembly algorithm and its applications to single-cell sequencing. Journal of Computational Biology 19: 455–477. 10.1089/cmb.2012.0021 22506599 PMC3342519

[B2] Belokobylskij SA (1989) The braconid wasps of the tribe Hormiini (Hymenoptera, Braconidae) from Australia. Entomologicheskoe Obozrenie 68: 376–392. [In Russian]

[B3] Belokobylskij SA (1993) On the classification and phylogeny of the braconid wasps of subfamilies Doryctinae and Exothecinae (Hymenoptera, Braconidae). Part I. On the classification, 2. Entomologicheskoe Obozrenie. 72: 143–164. [In Russian]

[B4] Belokobylskij SA Maeto K (2006) A new species of the genus *Parachremylus* Granger (Hymenoptera: Braconidae), a parasitoid of *Conopomorpha* lychee pests (Lepidoptera: Gracillariidae) in Thailand. Journal of Hymenoptera Research 15: 181–186.

[B5] Belokobylskij SA Manukyan AR (2024) First reliable fossil record of the subfamily Rhysipolinae (Hymenoptera: Braconidae): a new subgenus and species of the genus *Rhysipolis* Foerster, 1863 from Baltic amber. Zootaxa 5448: 591–600. 10.11646/zootaxa.5448.4.10 39646235

[B6] Belokobylskij SA Tobias VI (1986) Subfam. Doryctinae. In: Medvedev GS (Ed.) Opredelitel’ Nasekomych Evrospeiskoy Chasti SSSR, 3, Pereponchatokrylye, 4, 21–72. [In Russian]

[B7] Bolger AM Lohse M Usadel B (2014) Trimmomatic: a flexible trimmer for Illumina sequence data. Bioinformatics 30: 2114–2120. 10.1093/bioinformatics/btu170 24695404 PMC4103590

[B8] Branstetter MG Longino JT Ward PS Faircloth BC (2017) Enriching the ant tree of life: enhanced UCE bait set for genome‐scale phylogenetics of ants and other Hymenoptera. Methods in Ecology and Evolution 8: 768–776. 10.1111/2041-210X.12742

[B9] Buckingham GR Sharkey MJ (1988) Abdominal exocrine glands in Braconidae (Hymenoptera). Advances in Parasitic Hymenoptera Research 1988: 199–242.

[B10] Bushmanova E Antipov D Lapidus A Prjibelski AD (2019) rnaSPAdes: a de novo transcriptome assembler and its application to RNA-Seq data. GigaScience 8: giz100. 10.1093/gigascience/giz100 PMC6736328 31494669

[B11] Ceccarelli FS Sharkey MJ Zaldívar-Riverón A (2012) Species identification in the taxonomically neglected, highly diverse, Neotropical parasitoid wasp genus *Notiospathius* (Braconidae: Doryctinae) based on an integrative molecular and morphological approach. Molecular Phylogenetics and Evolution 62: 485–495. 10.1016/j.ympev.2011.10.018 22079550

[B12] Faircloth BC (2016) PHYLUCE is a software package for the analysis of conserved genomic loci. Bioinformatics 32: 786–788. 10.1093/bioinformatics/btv646 26530724

[B13] Gauld ID (1988) Evolutionary patterns of host utilization by ichneumonoid parasitoids (Hymenoptera: Ichneumonidae and Braconidae). Biological Journal of the Linnean Society 35: 351–377. 10.1111/j.1095-8312.1988.tb00476.x

[B14] Glenn TC Nilsen RA Kieran TJ Sanders JG Bayona-Vásquez NJ Finger JW Pierson TW Bentley KE Hoffberg SL Louha S Garcia-De Leon FJ del Rio Portilla MA Reed KD Anderson JL Meece JK Aggrey SE Rekaya R Alabady M Belanger M Winker K Faircloth BC (2019) Adapterama I: universal stubs and primers for 384 unique dual-indexed or 147,456 combinatorially indexed Illumina libraries (iTru & iNext). PeerJ 7: e7755. 10.7717/peerj.7755 PMC6791352 31616586

[B15] Harris RA (1979) A glossary of surface sculpturing. Occasional Papers in Entomology, State of California Department of Food and Agriculture 28: 1–31.

[B16] Hoang DT Chernomor O Von Haeseler A Minh BQ Vinh LS (2018) UFBoot2: improving the ultrafast bootstrap approximation. Molecular Biology and Evolution 35: 518–522. 10.1093/molbev/msx281 29077904 PMC5850222

[B17] Jasso‐Martínez JM Quicke DL Belokobylskij SA Meza‐Lázaro RN Zaldívar‐Riverón A (2021) Phylogenomics of the lepidopteran endoparasitoid wasp subfamily Rogadinae (Hymenoptera: Braconidae) and related subfamilies. Systematic Entomology 46: 83–95. 10.1111/syen.12449

[B18] Jasso-Martínez JM Quicke DL Belokobylskij SA Santos BF Fernández-Triana JL Kula RR Zaldívar-Riverón A (2022a) Mitochondrial phylogenomics and mitogenome organization in the parasitoid wasp family Braconidae (Hymenoptera: Ichneumonoidea). BMC Ecology and Evolution 22: 1–15. 10.1186/s12862-022-01983-1 35413835 PMC9006417

[B19] Jasso-Martínez JM Santos BF Zaldívar-Riverón A Fernández-Triana JL Sharanowski BJ Richter R Dettman JR Blaimer BB Brady SG Kula RR (2022b) Phylogenomics of braconid wasps (Hymenoptera, Braconidae) sheds light on classification and the evolution of parasitoid life history traits. Molecular Phylogenetics and Evolution 173: 107452. 10.1016/j.ympev.2022.107452 35307517

[B20] Katoh K Toh H (2008) Recent developments in the MAFFT multiple sequence alignment program. Briefings in Bioinformatics 9: 286–298. 10.1093/bib/bbn013 18372315

[B21] Lanfear R Frandsen PB Wright AM Senfeld T Calcott B (2017) PartitionFinder 2: new methods for selecting partitioned models of evolution for molecular and morphological phylogenetic analyses. Molecular Biology and Evolution 34: 772–773. 10.1093/molbev/msw260 28013191

[B22] Minh BQ Schmidt HA Chernomor O Schrempf D Woodhams MD von Haeseler A Lanfear R (2020) IQ-TREE 2: new models and efficient methods for phylogenetic inference in the genomic era. Molecular Biology and Evolution 37: 1530–1534. 10.1093/molbev/msaa015 32011700 PMC7182206

[B23] Muesebeck CFW (1958) New Neotropical wasps of the family Braconidae (Hymenoptera) in the U.S. National Museum. Proceedings of the United States National Museum 107: 405–461. 10.5479/si.00963801.108-3389.405

[B24] Quicke DL (2015) The Braconid and Ichneumonid Parasitoid Wasps: Biology, Systematics, Evolution and Ecology. John Wiley & Sons, Chichester, U.K. 10.1002/9781118907085

[B25] Quicke DLJ Belokobylskij SA Smith MA Rota J Hrcek J Butcher BA (2016) A new genus of rhysipoline wasp (Hymenoptera: Braconidae) with modified wing venation from Africa and Papua New Guinea, parasitoid of Choreutidae (Lepidoptera). Annales Zoologici 66: 173–192. 10.3161/00034541ANZ2016.66.2.003

[B26] Quicke DLJ Belokobylskij SA Braet Y van Achterberg C Hebert PDN Prosser SWJ Austin AD Fagan-Jeffries EP Ward DF Shaw MR Butcher BA (2020) Phylogenetic reassignment of basal cyclostome braconid parasitoid wasps (Hymenoptera) with description of a new, enigmatic Afrotropical tribe with a highly anomalous 28S D2 secondary structure. Zoological Journal of the Linnean Society 190: 1002–1019. 10.1093/zoolinnean/zlaa037

[B27] Scatolini D Penteado-Dias AM van Achterberg C (2002) *Pseudorhysipolis* gen. nov. (Hymenoptera: Braconidae: Rhysipolinae), with nine new species from Brazil, Suriname and Panama. Zoologische Mededelingen 76: 109–131.

[B28] Sharanowski BJ Dowling AP Sharkey MJ (2011) Molecular phylogenetics of Braconidae (Hymenoptera: Ichneumonoidea), based on multiple nuclear genes, and implications for classification. Systematic Entomology 36: 549–572. 10.1111/j.1365-3113.2011.00580.x

[B29] Sharkey MJ (1997) Key to New World subfamilies of the family Braconidae. In: Manual of the New World genera of the family Braconidae (Hymenoptera). International Society of Hymenopterists, Special Publ. 1, Washington, DC, 39–63.

[B30] Sharkey MJ Wharton RA (1997) Morphology & terminology. In: Manual of the New World genera of the family Braconidae (Hymenoptera). International Society of Hymenopterists Special Publ. 1, Washington, DC, 19–37.

[B31] Shaw MR (1983) On [One] evolution of endoparasitism: the biology of some genera of Rogadinae (Braconidae). Contributions of the American Entomological Institute 20: 307–328.

[B32] Shaw MR (2017) Further notes on the biology of *Pseudavgaflavicoxa* Tobias, 1964 (Hymenoptera, Braconidae, Rhysipolinae). Journal of Hymenoptera Research 54: 113–128. 10.3897/jhr.54.10789

[B33] Shaw MR Sims I (2015) Notes on the biology, morphology, nomenclature and classification of *Pseudavgaflavicoxa* Tobias, 1964 (Hymenoptera, Braconidae, Rhysipolinae), a genus and species new to Britain parasitizing *Bucculatrixthoracella* (Thunberg) (Lepidoptera, Bucculatricidae). Journal of Hymenoptera Research 42: 21–32. 10.3897/JHR.42.8935

[B34] Shimbori EM Souza-Gessner CSS Penteado-Dias AM Shaw SR (2017) A revision of the genus *Andesipolis* (Hymenoptera: Braconidae: Mesostoinae) and redefinition of the subfamily Mesostoinae. Zootaxa 4216(2): 101–152. 10.11646/zootaxa.4216.2.1 28183125

[B35] Shimbori EM Castañeda-Osorio R Jasso-Martínez JM Penteado-Dias AM Gadelha SS Brady SG Quicke DLJ Kula RR Zaldívar-Riverón A (2024) UCE-based phylogenomics of the lepidopteran endoparasitoid wasp subfamily Rogadinae (Hymenoptera: Braconidae) unveils a new Neotropical tribe. Invertebrate Systematics 38: IS24040. 10.1071/IS24040 39116275

[B36] Soltis PS Soltis DE (2003) Applying the bootstrap in phylogeny reconstruction. Statistical Science 18: 256–267. 10.1214/ss/1063994980

[B37] Spencer L Whitfield JB (1999) Revision of the Nearctic Species of *Rhysipolis* Förster (Hymenoptera: Braconidae). Transactions of the American Entomological Society (1890) 125: 295–324. http://www.jstor.org/stable/25078684

[B38] Tagliacollo VA Lanfear R (2018) Estimating improved partitioning schemes for ultraconserved elements. Molecular Biology and Evolution 35: 1798–1811. 10.1093/molbev/msy069 29659989 PMC5995204

[B39] Talavera G Castresana J (2007) Improvement of phylogenies after removing divergent and ambiguously aligned blocks from protein sequence alignments. Systematic Biology 56: 564–577. 10.1080/10635150701472164 17654362

[B40] Tobias VI (1964) Novye vidy I rod braconid (Hymenoptera, Braconidae) iz Tadzhikistana. Izvestiya Akademii Nauk Tadzhikskoi SSR 2: 58–65. [In Russian]

[B41] Tobias VI (1971) Review of the Braconidae (Hymenoptera) of the U.S.S.R. Proceedings of the All-Union Entomological Society 54: 156–268. [In Russian]

[B42] van Achterberg C (1995) Generic revision of the subfamily Betylobraconinae (Hymenoptera: Braconidae) and other groups with modified fore tarsus. Zoologische Verhandelingen 298: 1–242.

[B43] van Achterberg C (1988) Revision of the subfamily Blacinae Foerster (Hymenoptera: Braconidae). Zoologische Verhandelingen 64: 1–20.

[B44] Wharton RA (1993) Review of the Hormiini (Hymenoptera: Braconidae) with a description of new taxa. Journal of Natural History 27: 107–171. 10.1080/00222939300770061

[B45] Whitfield JB (1992) The polyphyletic origin of endoparasitism in the cyclostome lineages of Braconidae (Hymenoptera). Systematic Entomology 17: 273–286. 10.1111/j.1365-3113.1992.tb00338.x

[B46] Whitfield JB van Achterberg C (1987) Clarification of the taxonomic status of the genera *Cantharoctonus* Viereck, *Noserus* Foerster and *Pseudavga* Tobias (Hymenoptera: Braconidae). Systematic Entomology 12: 509–518. 10.1111/j.1365-3113.1987.tb00221.x

[B47] Whitfield JB Wagner DL (1991) Annotated key to the genera of Braconidae (Hymenoptera) attacking leafmining Lepidoptera in the Holarctic Region. Journal of Natural History 25: 733–754. 10.1080/00222939100770481

[B48] Whitfield JB Wharton RA (1997) Subfamily Hormiinae. In: Wharton RA Marsh PM Sharkey MJ (Eds) Manual of the New World Genera of the Family Braconidae (Hymenoptera).International Society of Hymenopterists, Special Publ. 1, Washington, DC, 285–302.

[B49] Yu DSK van Achterberg C Horstmann K (2016) Taxapad, Ichneumonoidea 2015. Database on flash-drive, Ottawa, Ontario. http://www.taxapad.com/

[B50] Zaldívar-Riverón A Mori M Quicke DL (2006) Systematics of the cyclostome subfamilies of braconid parasitic wasps (Hymenoptera: Ichneumonoidea): a simultaneous molecular and morphological Bayesian approach. Molecular Phylogenetics and Evolution 38: 130–145. 10.1016/j.ympev.2005.08.006 16213168

