# Supplementary figures and images for: ﻿Systematics of the braconid wasp subfamily Rhysipolinae (Hymenoptera, Braconidae) based on UCE data, with the description of a new Neotropical genus
Source: Zookeys. 2025 Apr 8;1234:67–87. doi: 10.3897/zookeys.1234.147859 (PMC12000810; doi:10.3897/zookeys.1234.147859)

1

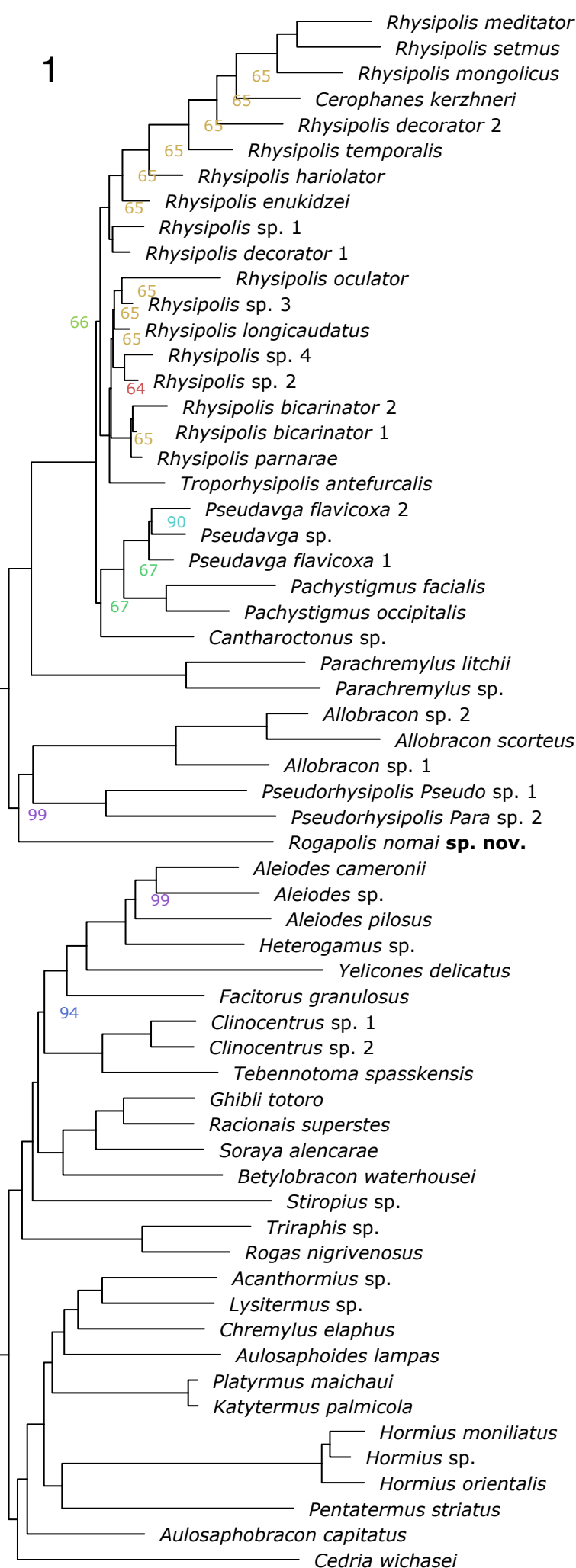

0.04

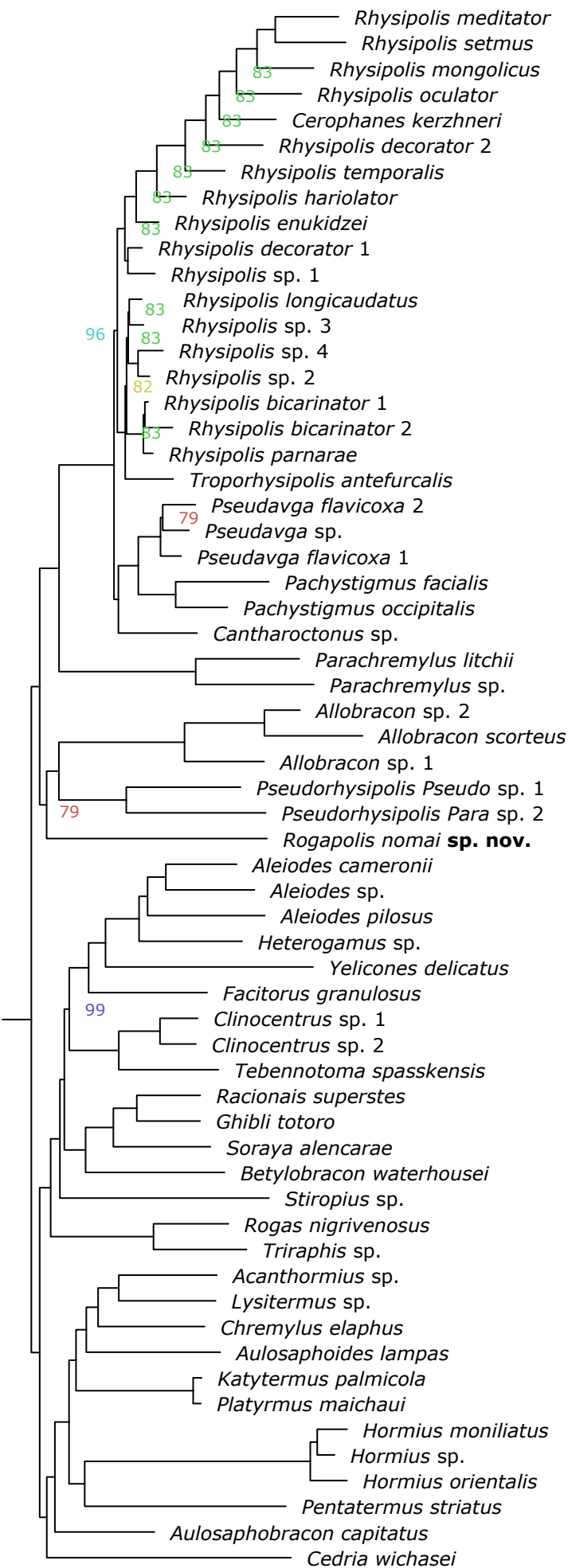

Supplement: Supplementary material 2 — ML phylograms derived from the 60% and 70% completeness matrices (1 and 2, respectively) [file zookeys-1234-067_article-147859__-s002.pdf]

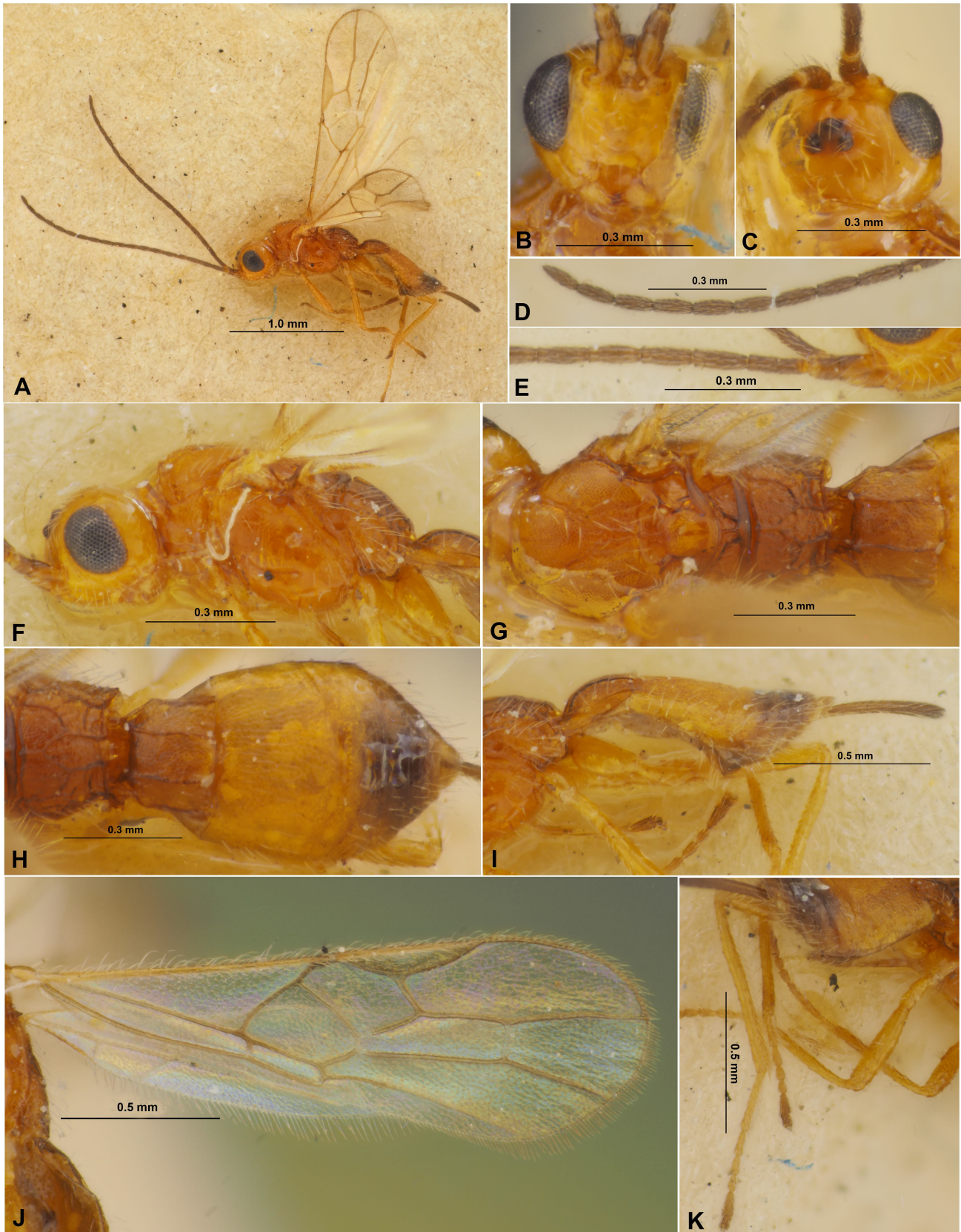

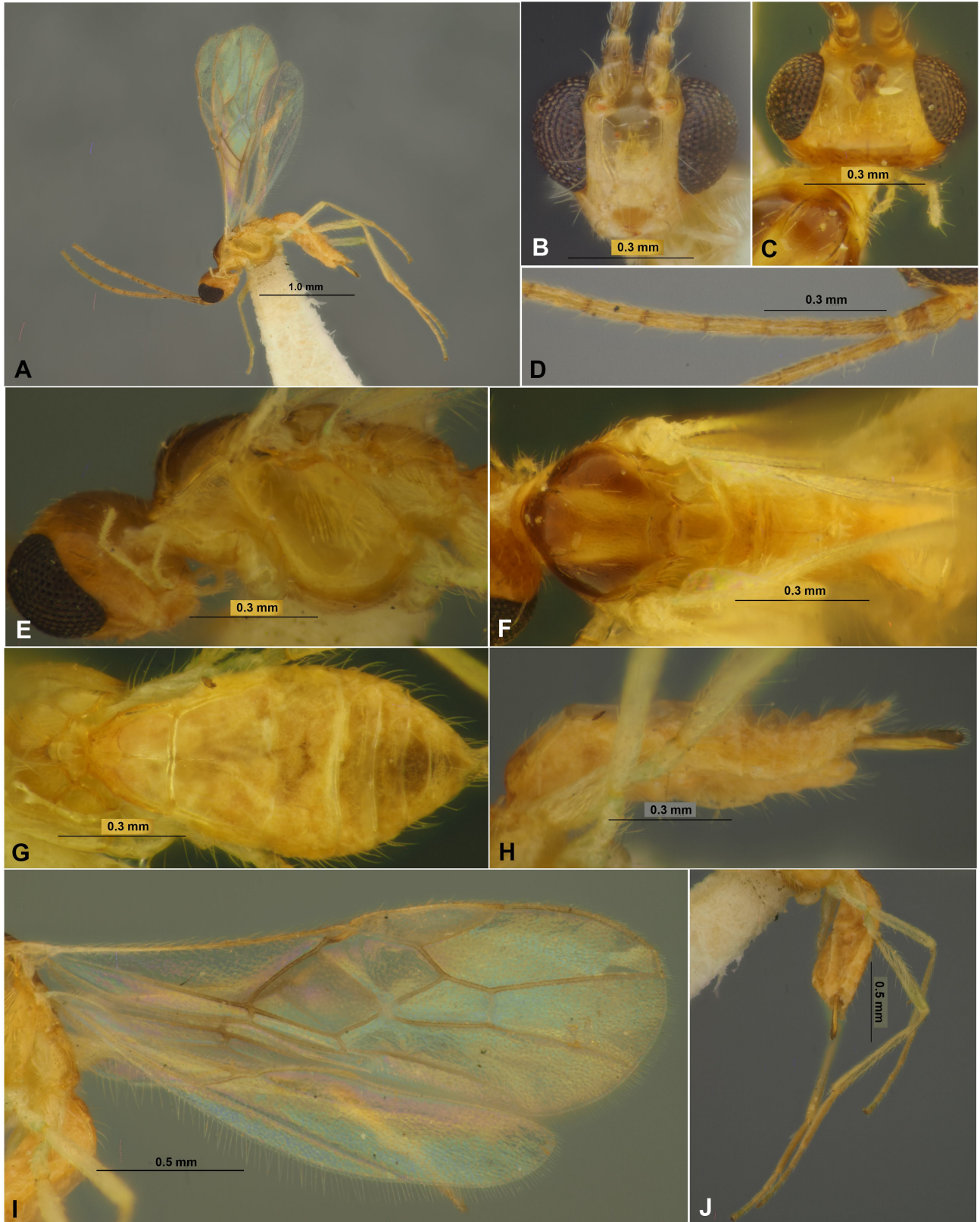

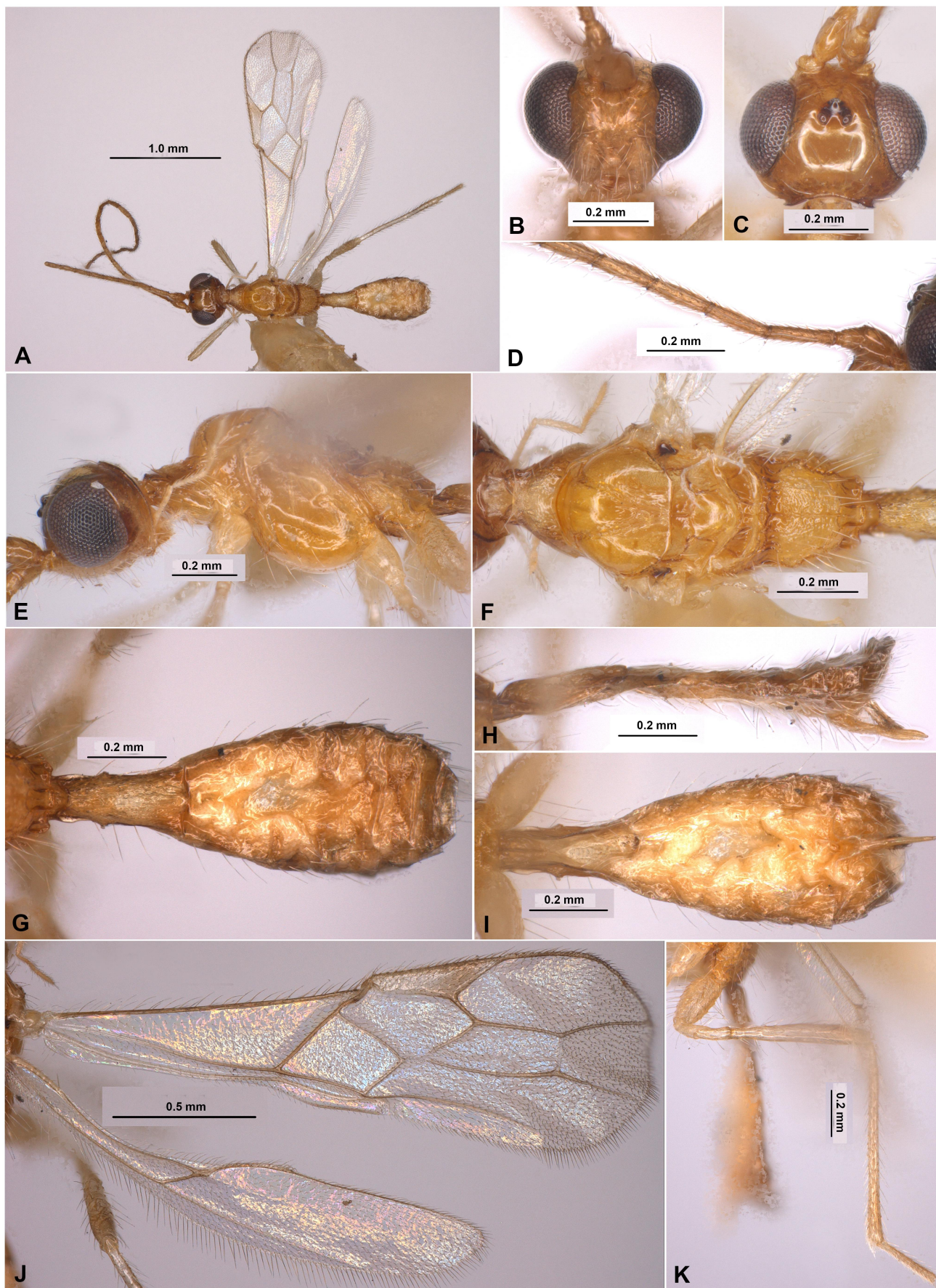

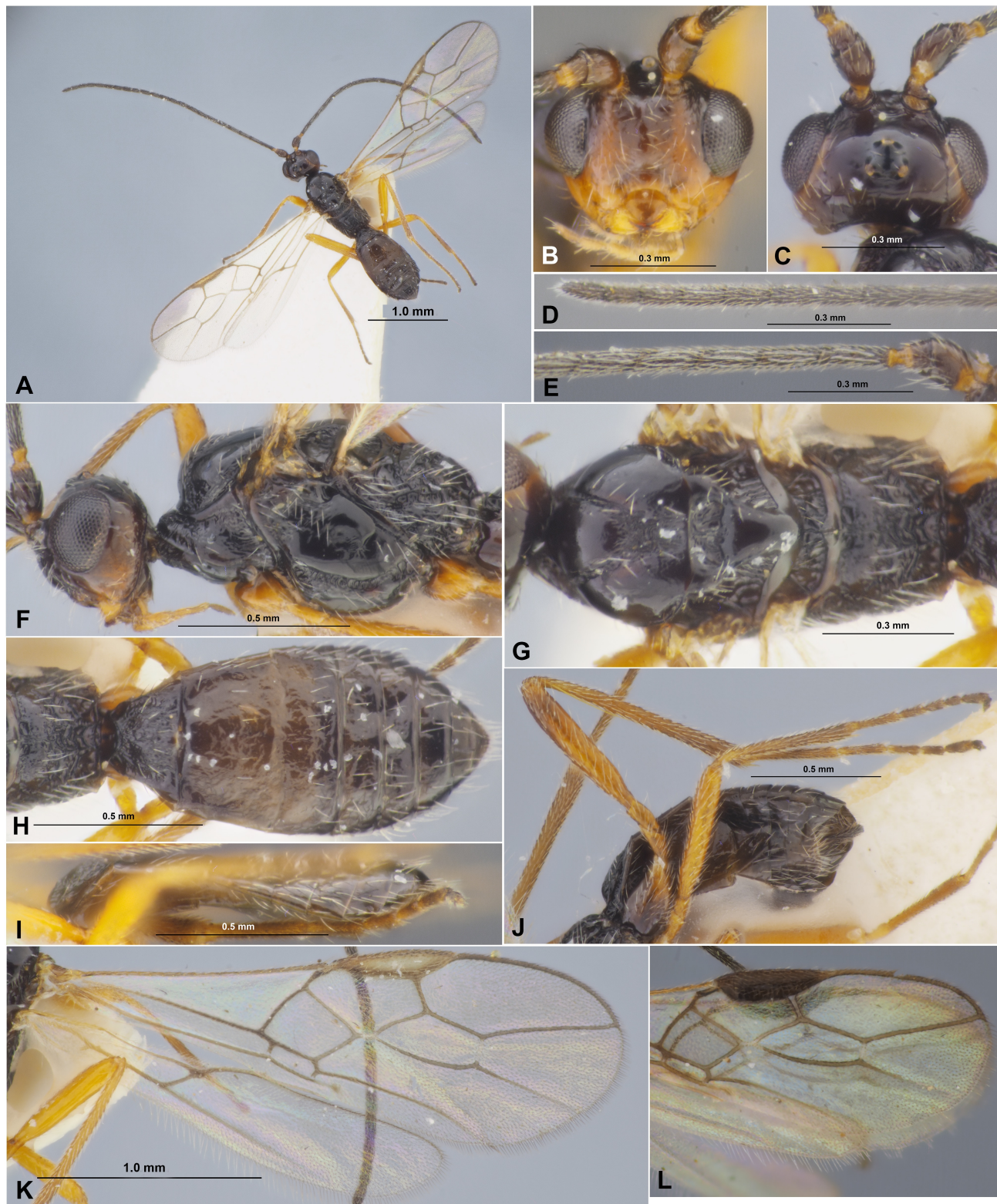

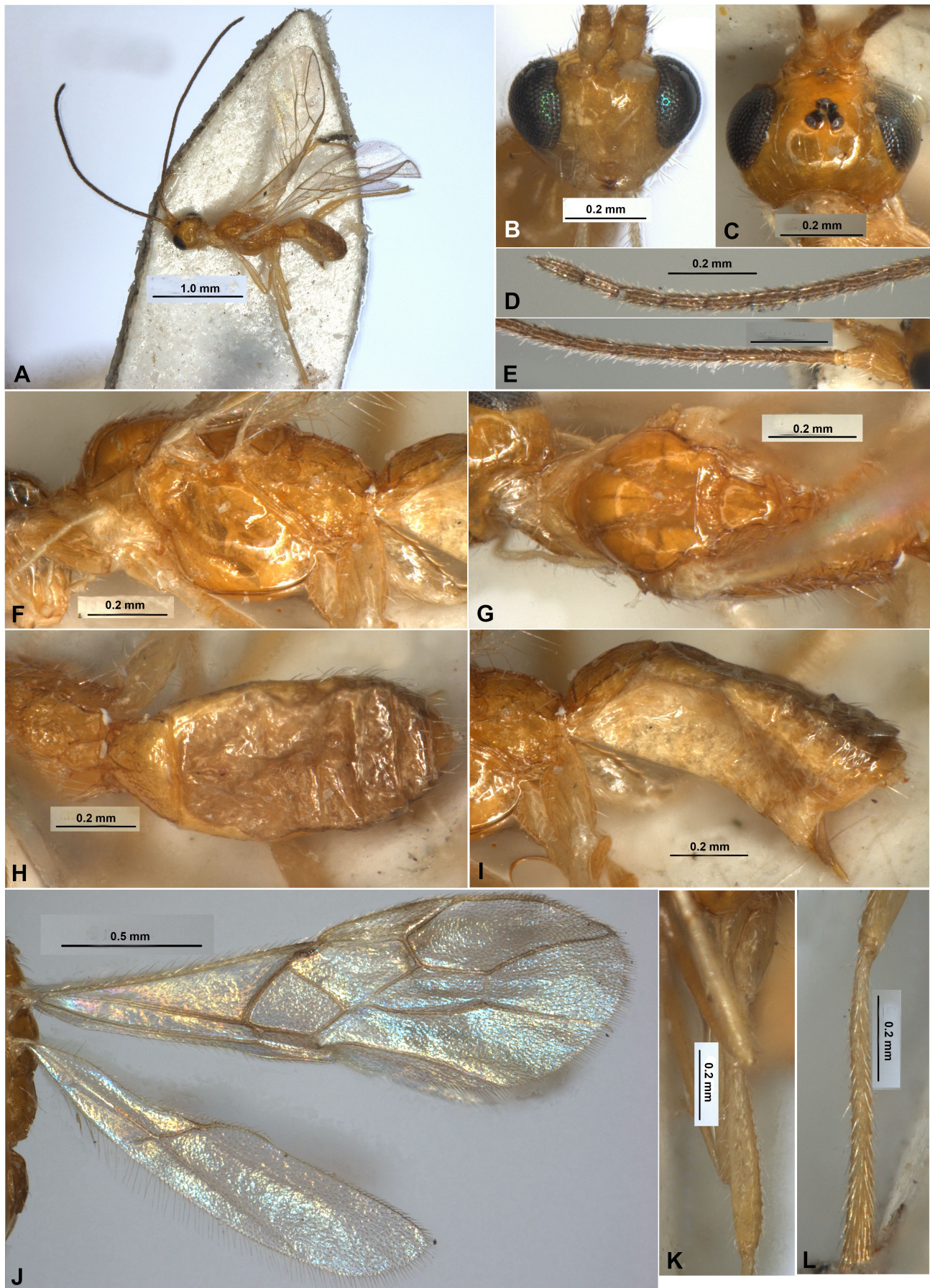

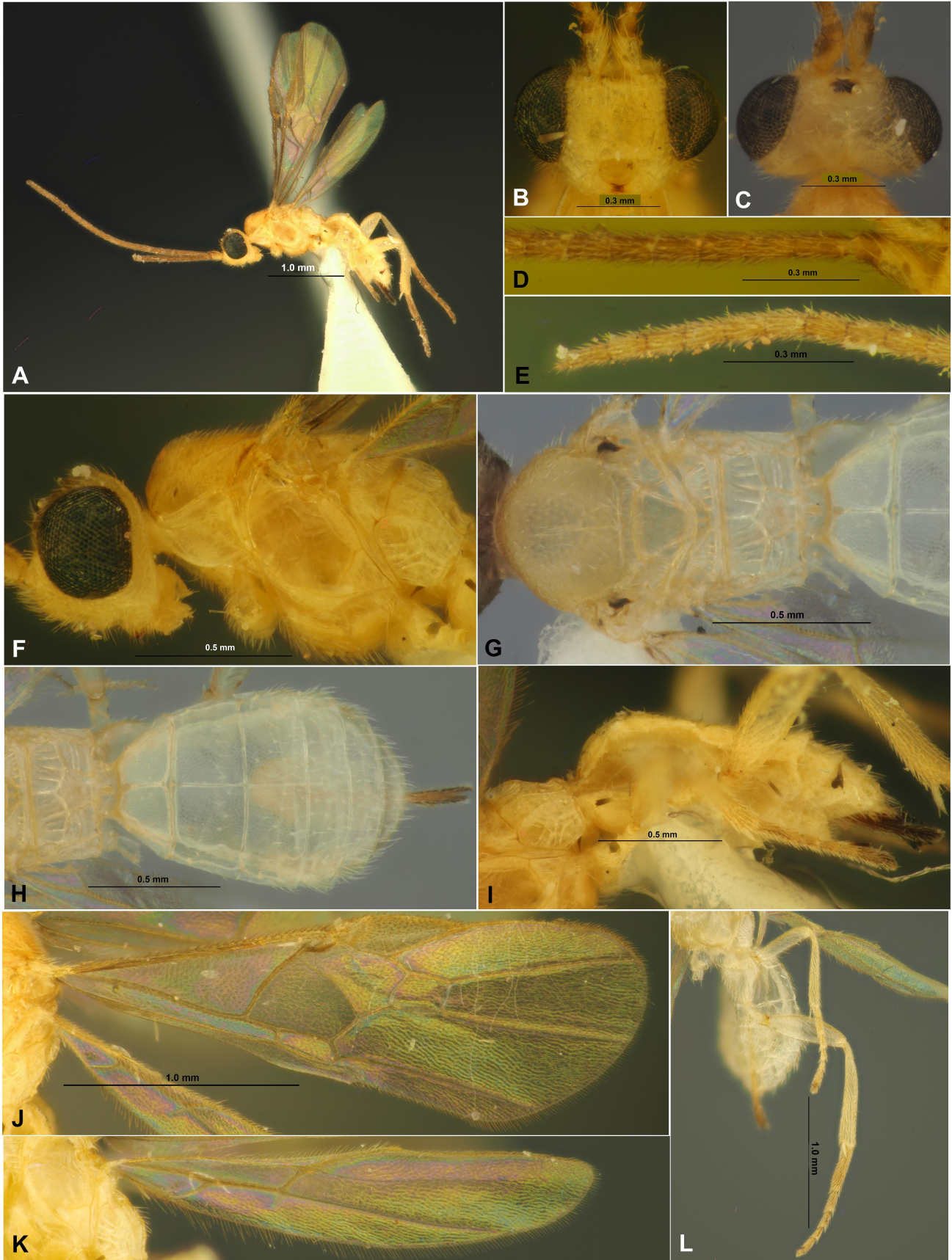

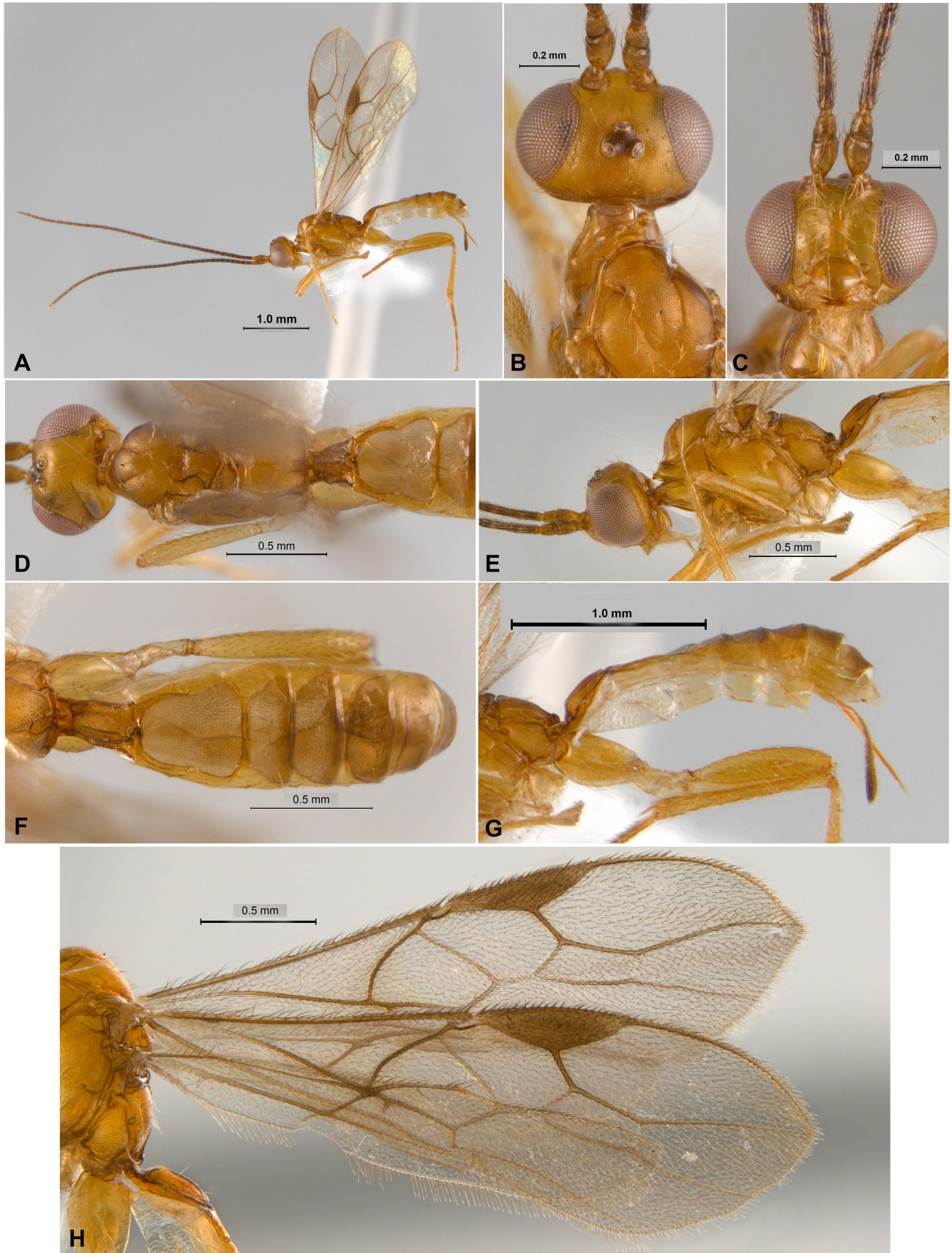

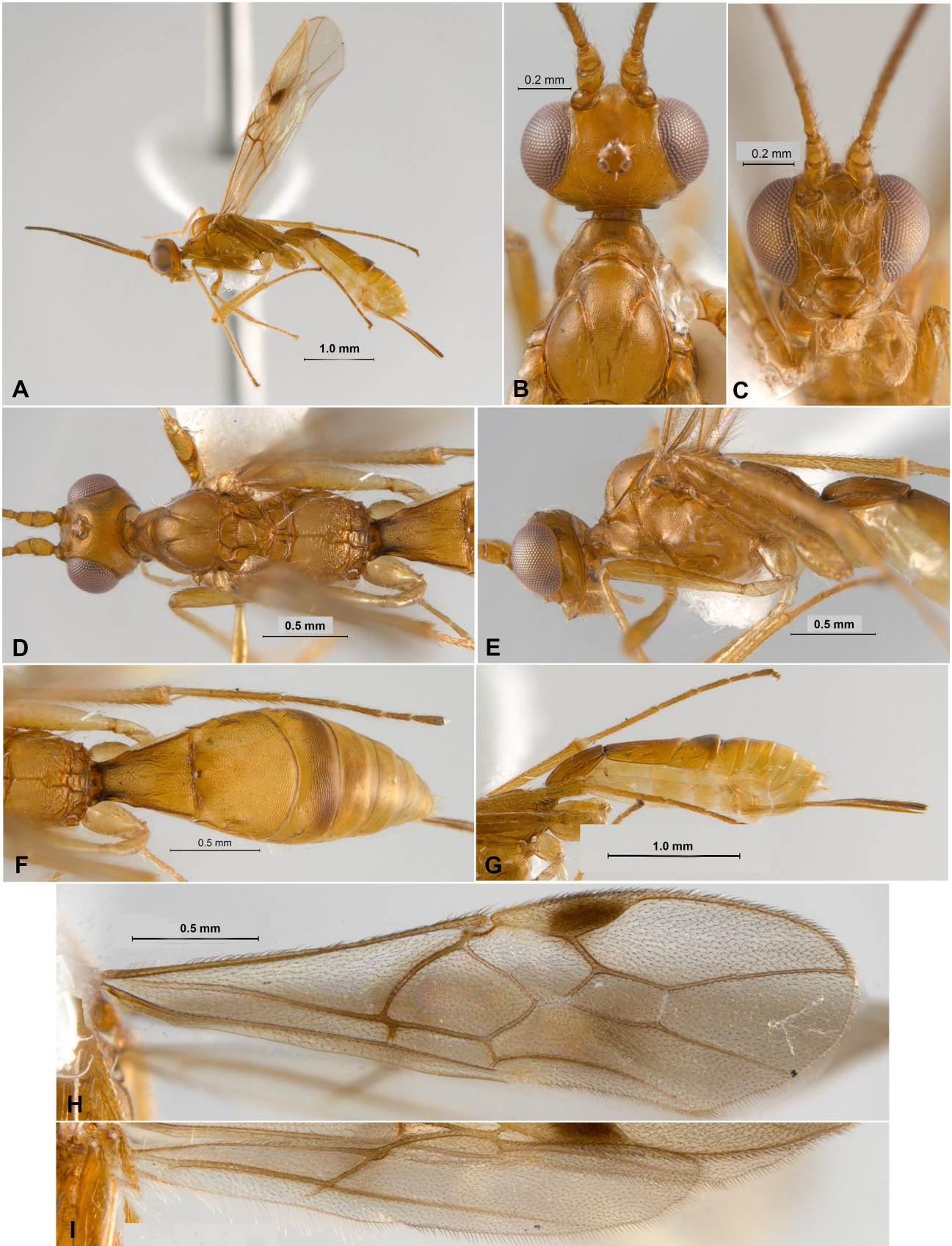

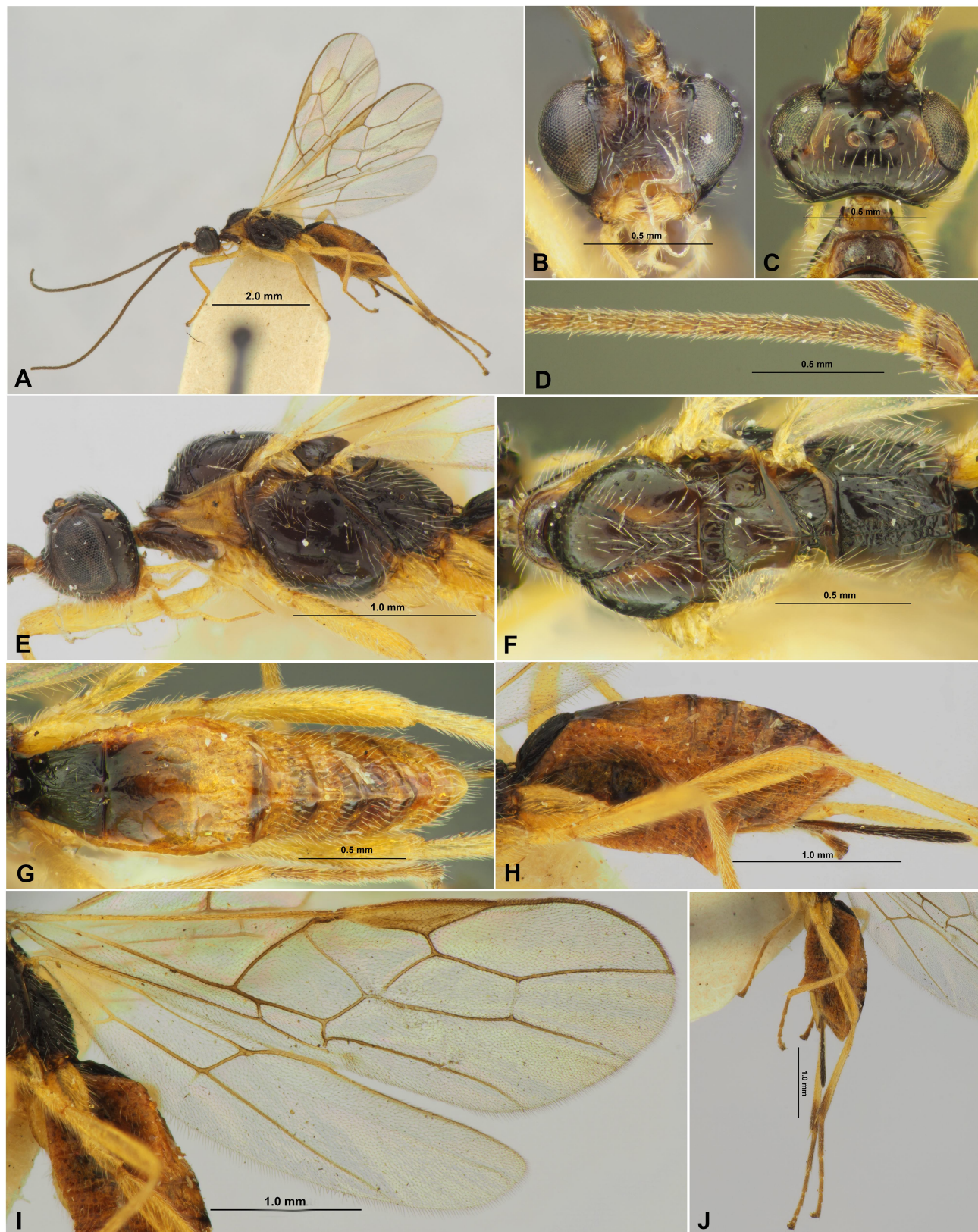

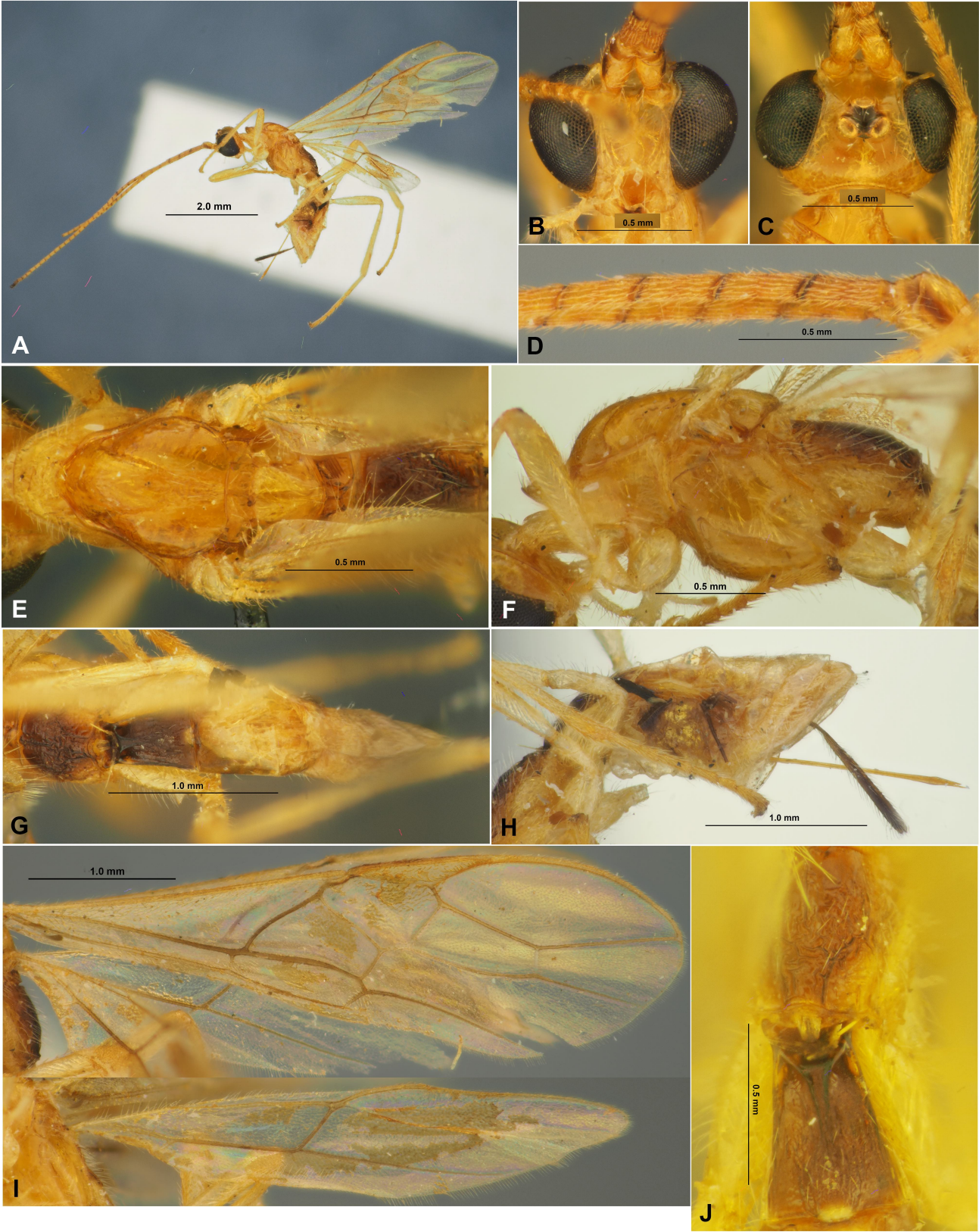

Supplement: Supplementary material 3 — Digital pictures of representative species belonging to all the rhysipoline genera recognised in this study except Rogapolis [file zookeys-1234-067_article-147859__-s003.pdf]
